# *Cornus mas* L. Extracts Exhibit Neuroprotective Properties, Further Enhanced by Metal-Bound Energy-Linked Organic Substrates

**DOI:** 10.3390/ijms26031159

**Published:** 2025-01-29

**Authors:** Georgios Lazopoulos, Sevasti Matsia, Marios Maroulis, Athanasios Salifoglou

**Affiliations:** Laboratory of Inorganic Chemistry and Advanced Materials, School of Chemical Engineering, Aristotle University of Thessaloniki, 54124 Thessaloniki, Greece; glazopou@cheng.auth.gr (G.L.); srmatsia@cheng.auth.gr (S.M.); info@modernanalytics.gr (M.M.)

**Keywords:** *Cornus mas* L. extracts, gene expression, metal ions, oxidative stress, neuroprotection, neurodegeneration therapeutics

## Abstract

Neurodegenerative diseases have been increasingly plaguing the global population, with attempts to understand their etiopathogenesis and pursue therapeutics being at the forefront of multidisciplinary efforts. To that end, research was launched in our lab, based on natural products and bioessential metal ion complex forms to peruse their antioxidant and neuroprotective potential at the cellular level. To that end, the bioactivity profile of optimized *Cornus mas* L. extracts and supplemented mixtures thereof with soluble-bioavailable well-characterized hybrid materials, Zn(II)-Cit and V(IV)-Cit, was investigated. In vitro experiments on sensitive brain tissue cell lines (N2a58, SH-SY5Y) showed that the extracts and the metal complexes were atoxic (morphology, proliferation, chemotacticity) in a concentration-dependent manner. Subsequently, the antioxidant potential of all materials was examined, with H_2_O_2_ as the oxidizing agent, thereby revealing through viability and reactive oxygen species (ROS) visualization significant antioxidant activity, while specific genes (*NFE2L2*, *Hmox1*, *GCLM*) were crucial in divulging mechanistic aspects of the antioxidation. Concurrently, the anti-inflammatory activity was evaluated through gene expression (*TNF-a*, *IL-6*), with Zn(II) bioavailability projecting intracellular levels linked to the observed sustainable activity. The collective bioactivity profile of the extracts and Zn(II)-Cit reveals significant neuroprotective properties, thereby meriting development of new naturally-based neutraceuticals that proactively avert neuropathological aberrations.

## 1. Introduction

Neurodegeneration is exemplified through a heterogeneous family of chronic, progressively worsening disorders characterized by gradual neuronal dysfunction and death, effectively compromising motor and cognitive function. The most common neurodegenerative diseases include Alzheimer’s disease, Parkinson’s disease, amyotrophic lateral sclerosis, multiple sclerosis, and Huntington’s disease [1,2]. As the global human population ages, the number of cases of neurodegenerative diseases is projected to grow, from 13.5 million in 2000 to 21.2 million in 2025 and 36.7 million in 2050 [3]. There are various factors leading to the pathogenesis of these diseases; however, mounting evidence suggests that ROS (reactive oxygen species) play a critical role in the onset and progression of the respective pathological conditions [4]. Moderate ROS concentration can act as a regulator of physiological cellular processes, such as redox signaling transduction, gene expression, and receptor activation, thus leading to tissue turnover and cell proliferation [5]. On the other hand, when indigenous antioxidant systems cannot modulate and control ROS accumulation due to an increase in the production of free radicals, oxidative stress sets in [6]. Oxidative stress can influence all biomacromolecules, causing lipid peroxidation, protein misfolding and aggregation, RNA mistranscription, and DNA damage. It seems that ROS toxicity is mediated by post-translational modifications of signaling molecules and/or hallmark proteins of neurodegeneration, while at the same time, free radicals pass freely through the plasma membrane, resulting in cell leakage and death [7]. In the face of such adversity, the brain is particularly vulnerable to oxidative damage because it contains a high concentration of radical-susceptible polyunsaturated fatty acids, while at the same time, high levels of redox-active iron can act as a prooxidant, thereby contributing to the induction of autophagic cell death. Furthermore, lipid peroxidation of unsaturated fatty acids leads to the production of highly toxic compounds, including aldehydes or dienals, which can cause neuronal apoptosis. Aside from the aforementioned, the brain is a major metabolizer of oxygen (with a consumption of nearly 20% of the consumption of the entire body), while at the same time, the protective antioxidant mechanism is relatively inefficient [8]. In fact, the brain contains low levels of glutathione, almost no catalase, and compared to the liver, reduced concentrations of glutathione peroxidase and vitamin E [9].

Natural products are currently a subject of great interest in the pharmaceutical, health, food, and cosmetic industry owing to their diverse biological activities. The plant kingdom remains an insufficiently known source of bioactive natural compounds with various beneficial activities, such as anti-cancer, immunostimulant, anti-inflammatory, antioxidant, neuroprotective, and hepatoprotective [10]. The most studied forms of natural products are extracts. The advantage of an extract, compared to isolated molecules, is the possible synergistic effect that the plethora of molecules in the extract might have, thus augmenting its collective beneficial effects. However, through this approach, due to inadequate fractionation processes, it is challenging to identify individual molecules responsible for the observed phenotype and macroscopic activity of the extracts [11]. *Cornus mas* L., also known as dogwood, is a plant of the Cornaceae family that grows in Eastern Europe and the Middle East [12]. The plant is widely used in folk medicine against various diseases, such as diarrhea, hemorrhoids, diabetes, and sore throat. Many authors over the years have attributed these properties to the high concentration of anthocyanins due to their physiological role in plants. Apart from anthocyanins, the chemical composition of the specific fruit can be divided into four main structural groups, i.e., iridoids, phenolic acids, flavonoids, and tannins. The quantity of these compounds is greatly dependent on the plant genotype, cultivation conditions, and ripeness of the fruit [13,14]. These components have been linked to many health-promoting effects, either as isolated compounds or synergistic combinations thereof. The main properties of these compounds are (a) antioxidant, emanating from the present polyphenols; (b) anti-inflammatory, anti-osteoporotic, and antiglaucomic due to the presence of loganic acid; (c) hepatoprotective from the anthocyanins and iridoids; as well as (d) anti-atherosclerotic and antidiabetic from whole fruit extracts [15].

Concurrently, metal ions are a necessary part of human physiology, the deficiencies or paucity of which can result in pathological aberrations. Zinc, in particular, is a known antioxidant in the immune system, with zinc supplementation also blocking NF-κB activity, a known mediator of pro-inflammatory gene induction. On the other hand, vanadium, due to its pluripotent coordination chemistry, the various physiologically relevant oxidation states of the metal ion, and its flexibility in promoting complexation chemistries, can act as a malleable therapeutic agent, depending on the desired medicinal characteristics [16,17,18,19]. Along the same lines, citric acid is an α-hydroxytricarboxylic acid found in the metabolism of all aerobic organisms, as a key energy intermediate in the citric acid cycle (Krebs cycle), widely used for its antioxidant capacity in pharmaceutical, food, and beverage industries. At the same time, citric acid can act as a very soluble and effective metal ion chelator, thus ending up being a very good option as a ligand for bioessential metal ions [20,21]. On the other hand, the supplementation of bioavailable metals, especially in the case of zinc ions, might provide certain challenges since there is well-established evidence detailing the dynamic interplay between zinc and calcium ions in cell physiology that could result in irregular protein aggregation [22,23] and bear down on the integrity and survival of cells. At the same time, the introduction of metal ions in physiological fluids could have an influence on the ionic strength of the medium, thereby influencing the type of aggregation of subcellular targets related to neurodegenerative diseases [24].

On the basis of the aforementioned grounds and specified factors, research has been launched in our lab to seek, identify, study, delineate, and formulate hybrid formulations of natural product materials poised to contribute to the amelioration of human neuropathological conditions and/or contribute to the proactive aversion of the onset or retardation of oxidative stress-induced neurodegenerative events. Cognizant of the physicochemical power of the above-described natural agents of inorganic or organic nature, carefully designed and appropriately configured abiotic and biological forms of such agents were employed. To that end, an attempt was made in the herein-described work to delineate the effectiveness of *Cornus mas* L. extracts against oxidative stress as a major contributor to neurodegeneration, with comprehensive studies poised to discover, define, and test in vitro well-configured hybrid formulations of the extracts supplemented by bioinorganic complex forms of zinc and vanadium (both biologically relevant and essential metal ions in biological systems in nature), collectively presenting a comprehensive neuroprotective profile. The generated profile(s) of neuronal cell (a)toxicity, antioxidant activity, anti-inflammatory activity, and cellular mechanism of action, as well as the ability of the hybrid bioinorganic materials to enhance such actions, provide a platform of natural product derivatives with merit as nutraceutical supplements, acting against oxidative stress and potentially serving as neuroprotectants.

## 2. Results

### 2.1. Hybrid Material Synthesis

#### 2.1.1. Zn(II)-Cit

**Figure 1:**
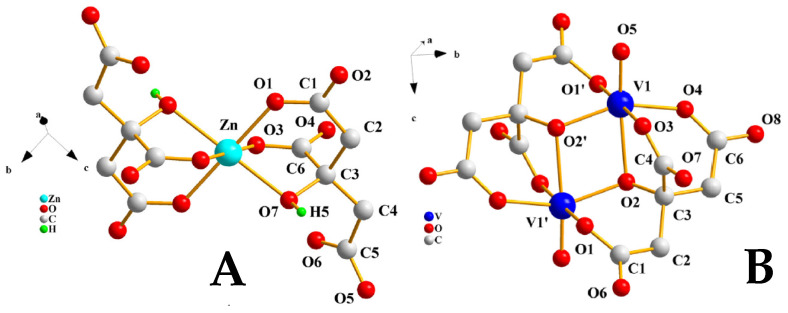
The 3D structure of the anions in (**A**) Zn(II)-Cit, and (**B**) V(IV)-Cit hybrid complex materials.

Zn(II)-Cit ((NH_4_)_4_[Zn(C_6_H_5_O_7_)_2_]) crystalline material was synthesized, isolated, and positively identified analytically, spectroscopically, and crystallographically prior to use. The yield of the synthesis reaction of the hybrid material was 58.0%. The 3D crystallographic structure of Zn(II)-Cit in the solid state is provided in Figure 1A, showing the Zn(II) center being bound to two triply deprotonated citrato ligands, specifically two carboxylato and one alkoxido oxygens from each ligand, thus giving rise to a distorted octahedral geometry and promoting the stability of the complex due to formation of a five-membered metallacycle ring. The carboxylate group that does not bind to Zn(II) is a terminal one, dangling away from the metal ion [25]. The balanced stoichiometric chemical reaction of the synthesis is depicted below:


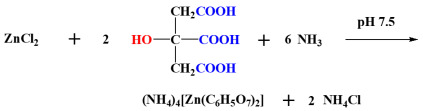


#### 2.1.2. V(IV)-Cit 

V(IV)-Cit (Na_4_[V_2_O_2_(C_6_H_4_O_7_)_2_]·12H_2_O) crystalline material was isolated and positively identified analytically, spectroscopically, and crystallographically prior to use. The yield of the reaction was 77.0%. The 3D structure in the solid state is shown in Figure 1B, involving a V_2_O_2_ core assembly with two V=O groups linked to the alkoxido oxygens of the citrato ligands. The two citrato ligands are fully deprotonated, and each vanadium ion is bound to three carboxylato oxygens (one from the first citrato ligand and two from the second one), ultimately achieving octahedral coordination [25]. The balanced stoichiometric chemical reaction of the synthesis is depicted below:


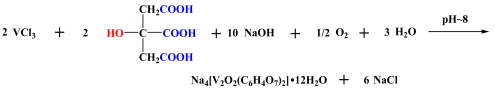


### 2.2. Total Phenolic Content (TPC) and Ferric Reduction Antioxidant Power (FRAP) Assays

The antioxidant capacity of *Cornus mas* L. (hereafter dubbed *Cornus*) aqueous extracts was evaluated through the TPC and FRAP methods. The amount of total phenols, as shown in Table 1, was found to be 6.48 ± 0.09 mg gallic acid equivalents (GAE)/g dry extracts. The antioxidant capacity related to *Cornus* extracts to reduce iron activity (Table 1) is 9.54 ± 0.04 mg ascorbic acid equivalents (AAE)/g dry extracts. The overall data are in consonance with the extraction procedures of *Cornus* extracts reported in the literature [26], giving total phenolic values of ~1500 mg GAE/100 g dry fruit. The findings are comparable and close to that mentioned in the literature for methanolic extracts of Cornelian cherry fruits [27,28].

### 2.3. In Vitro Cytotoxic Profile 

**Figure 2:**
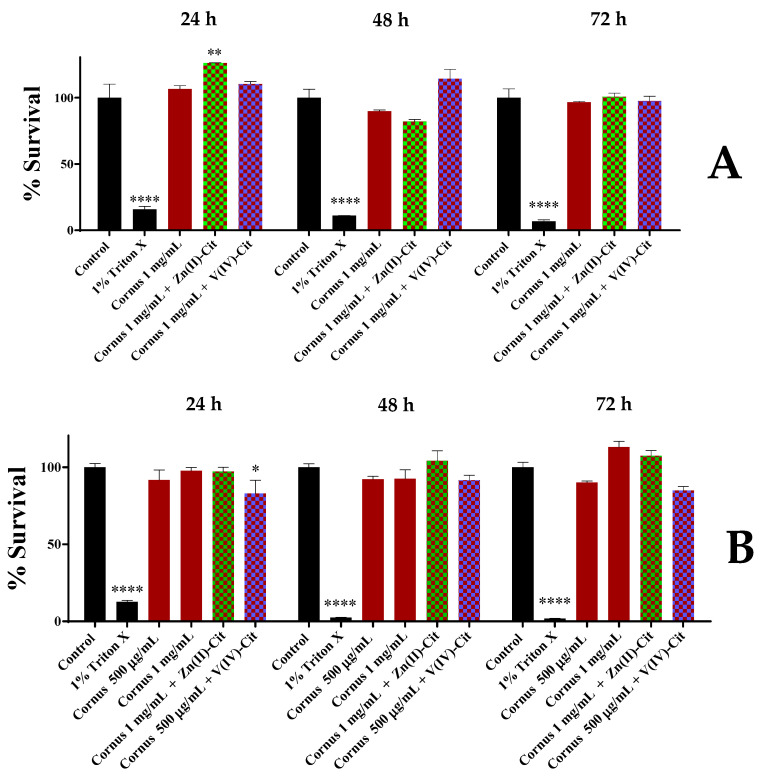
Survival percentage of (**A**) N2a58 or (**B**) SH-SY5Y cells, after treatment with the *Cornus mas* L. extracts at 1 mg/mL, with and without supplementation of Zn(II)-Cit 100 μM or V(IV)-Cit 10 μM, for 24, 48 and 72 h, compared to the untreated sample (control). Significance levels were assessed as follows: * *p* < 0.05 (significant), ** *p* < 0.01 (highly significant), *** *p* < 0.001 (extremely significant) and **** *p* ≤ 0.0001 (extremely significant).

To delineate the in vitro cytotoxicity profile of the extracts, N2a58 and SH-SY5Y neuronal cell cultures were treated with various concentrations of the extracts (from 10 pg/mL to 10 mg/mL) over various timeframes (24, 48, and 72 h). The timeframes were chosen so that the limits of cytotoxicity could be reached in biologically useful concentrations. In each case, the untreated cells are shown as (negative) control, while 1% Triton X-100 was used as positive control. The survival percentage of both cell lines is shown in the Supplementary Material (Figure S1). In both cell lines, the viability of the cells is not compromised until very high concentrations (>1 mg/mL) are reached. Furthermore, the cytotoxicity profile of Zn(II)-Cit and V(IV)-Cit was studied under similar conditions at various concentrations, extending into the range from 1 nM to 1 mM in the case of Zn(II)-Cit and in the range 1 μM to 100 μM for V(IV)-Cit and various timeframes (24, 48, 72 h). The results for both cell lines are shown in the Supplementary Material (Figures S2 and S3). In the case of Zn(II)-Cit, it does not reduce the viability of N2a58 cells under the conditions investigated (up to 1 mM for 72 h), whereas the viability of SH-SY5Y cell cultures is not influenced up to 100 μM. In the case of V(IV)-Cit, in the N2a58 cell line culture, the viability of the cells is not affected up to the concentration of 10 μM, over all timeframes investigated. Also, V(IV)-Cit appears to reduce the viability of the SH-SY5Y cell line culture at very low concentrations, with 1 μM being the limit of the absence of the viability impairment. From these studies, the maximum concentrations of all three materials, where the viability of cells is not impaired, were determined, and the combination of the extracts with the hybrid materials was also studied. The survival percentages of the combinations (extracts with hybrid material) in the presence of the appropriate controls for both cell lines are shown in Figure 2. The viability results of N2a58 cell cultures for the combination of the extracts with the hybrid materials show that there is no toxicity resulting from the supplementation. In an analogous fashion, in the case of SH-SY5Y cell cultures, the combination of atoxic concentrations of the extracts and Zn(II)-Cit (100 μΜ) does not reduce the viability of the cells. On the other hand, in the case of V(IV)-Cit, there is a reduction in viability of the mixture of atoxic concentrations, thus prompting us to select a reduced concentration (500 nΜ) of that material to achieve and maintain atoxicity.

In addition, further investigation of the cytotoxicity profile of the extracts and the hybrid materials included morphological studies at concentrations exhibiting toxicity or showing no toxicity as that was exemplified through the aforementioned viability experiments. Cell morphology at different concentrations was pursued, with the results shown in the Supplementary Material (Figures S4 and S5) for the extracts, (Figures S6 and S7) for Zn(II)-Cit, and (Figures S8 and S9) for V(IV)-Cit, in the case of the N2a58 and the SH-SY5Y cell cultures, respectively. The morphology of both cell lines in the presence of the extracts is consistent with the viability results, while in the presence of Zn(II)-Cit, 1 mM of the metal–organic complex species morphologically alters both cell lines, despite the fact that N2a58 cell cultures exhibit no impairment of their viability. 

**Figure 3:**
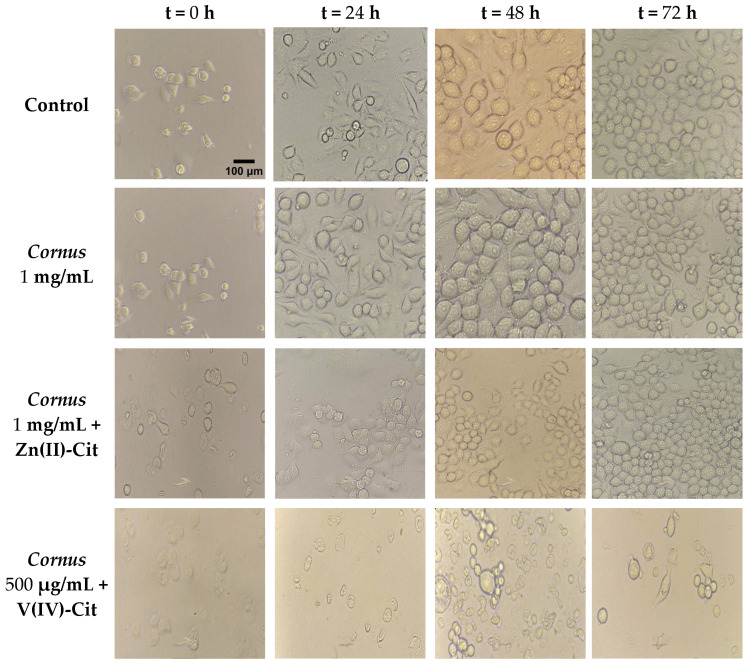
Morphological studies of N2a58, following treatment with a combination of *Cornus mas* L. extracts 1 mg/mL and 100 μM Zn(II)-Cit or extracts 500 μg/mL and 10 μM V(IV)-Cit for 24, 48, and 72 h, compared to the untreated sample (control).

Finally, V(IV)-Cit does not confer any morphological alteration upon either of the cell lines at concentrations where viability is not impaired. The morphological studies of the supplementation of *Cornus* extracts with Zn(II)-Cit and V(IV)-Cit materials on the N2a58 cell line cultures are shown in Figure 3, while the same studies for SH-SY5Y cell line cultures are shown in Figure S10. In the case of the N2a58 cells, zinc supplementation does not have a significant morphological impact on cells, whereas vanadium supplementation alters the morphology of the cells, thus leading to smaller and more aggregated cells. On the other hand, neither Zn(II) nor V(IV) complex supplementation seems to have an effect on SH-SY5Y morphology under the employed conditions.

**Figure 4:**
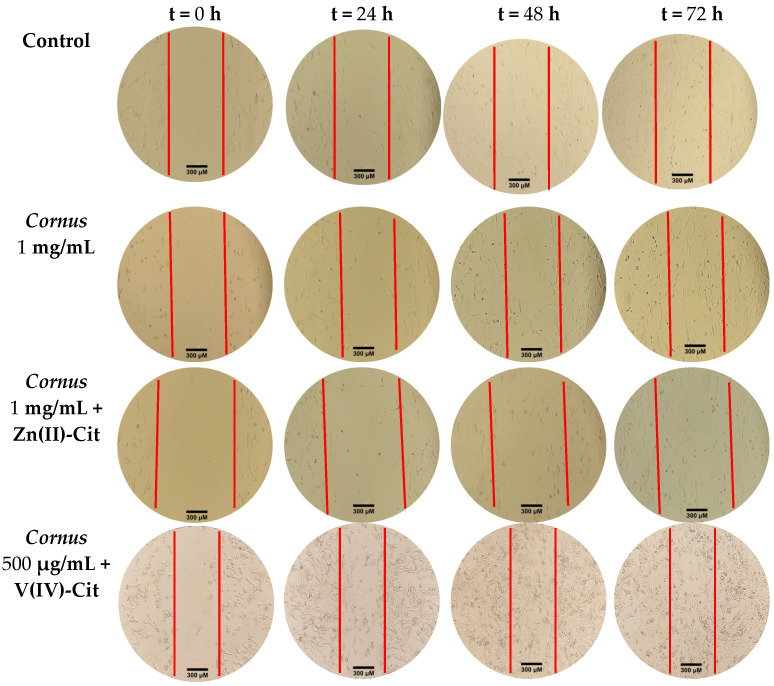
Chemotacticity studies using the wound-healing assay on SH-SY5Y cells, following treatment with a combination of *Cornus mas* L. extracts at 1 mg/mL and 100 μM Zn(II)-Cit or extracts at 500 μg/mL and 500 nM V(IV)-Cit for 24, 48, and 72 h, compared to the untreated sample (control).

The last parameter, which was taken into account regarding the cytotoxicity of the extracts and the hybrid materials, is chemotacticity and its changes compared to the untreated cells. In Figure 4, what is shown is the time-dependent and treatment-dependent wound-healing assay of SH-SY5Y cell cultures involving the mixture of the extracts with the hybrid supplements (Zn(II)-Cit or V(IV)-Cit). The corresponding pictures of treatments of separate extracts and hybrid materials are provided in the Supplementary Material (Figures S11 and S12). The speed of migration in each case was calculated and is provided in Table 2, expressed as the distance in mm that the cells have migrated per hour.

In the case of Zn(II)-Cit supplementation of the extracts, the migration speed is similar to the Zn(II)-Cit treatment itself. At the same time, V(IV)-Cit has no effect on the migration speed of the cells, whereas combined treatment involving both the extracts and vanadium leads to a slight decrease in the migration speed. It should be mentioned that during the implementation of the wound-healing assay in N2a58 cell line cultures, right after the scratch and the medium change, cells away from the scratch were observed to depart from their position, adhering at random positions in the middle of the scratch, thus negating the purpose of the specific experiment.

### 2.4. Antioxidant Studies

#### 2.4.1. Oxidative Stress Through Viability Assay

**Figure 5:**
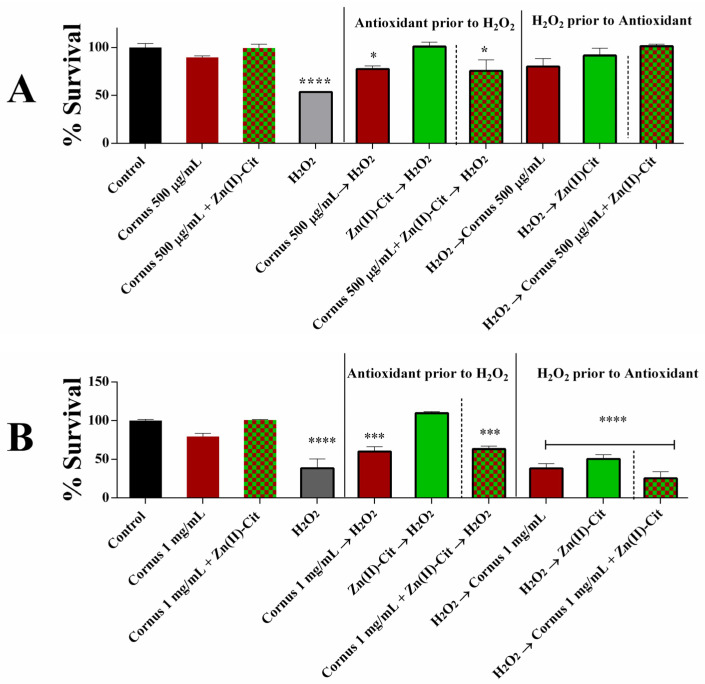
Survival percentage of (**A**) N2a58 cells, following treatment with *Cornus mas* L. extracts (500 μg/mL), Zn(II)-Cit (100 μM) or the mixture thereof, prior to or after treatment with H_2_O_2_ (500 μM), with the corresponding controls, compared to the untreated sample (control); (**B**) SH-SY5Y following treatment with the *Cornus mas* L. extracts (1 mg/mL), Zn(II)-Cit (100 μM) or the mixture thereof, prior to or after treatment with H_2_O_2_ (250 μM), with the corresponding controls, compared to the untreated sample (control). Significance levels were assessed as follows: * *p* < 0.05 (significant), ** *p* < 0.01 (highly significant), *** *p* < 0.001 (extremely significant) and **** *p* ≤ 0.0001 (extremely significant).

Following the determination of the complete cytotoxicity profile, the antioxidant activity of the *Cornus mas* L. extracts and the employed hybrid materials were examined. As a model for oxidative stress, H_2_O_2_ was chosen as an inducer at an appropriate concentration (500 μM) that promotes partial cell death. The viability studies of both N2a58 and SH-SY5Y cells under oxidative stress, in the presence of the extracts and Zn(II)-Cit as supplementation, are shown in Figure 5, while the viability studies of the extracts supplemented with V(IV)-Cit are provided in the Supplementary Material (Figure S13). 

For the N2a58 cell line culture, cell treatment with the extracts reveals antioxidant activity against oxidative stress, both when the extracts are added prior to or after the H_2_O_2_ treatment. Similarly, the Zn(II)-Cit hybrid material exhibits high antioxidant activity, fully neutralizing the toxicity of H_2_O_2_ treatment, either as a pre- or a post-treatment event. For the mixture of both Zn(II)-Cit and the extracts, there is significant antioxidant activity, with post-treatment being marginally better. In the case of V(IV)-Cit, pre-treatment of cells with either the hybrid material or the mixture of the hybrid material and the extracts reflects higher toxicity than the H_2_O_2_ alone, while post-treatment results show limited antioxidant activity in both cases.

Looking at the SH-SY5Y cell culture results, extract pre-treatment reveals antioxidant activity against H_2_O_2_-induced oxidative stress, while under the same conditions, Zn(II)-Cit shows full protection of the cells against ROS. At the same time, Zn(II) supplementation has no additional impact on the viability of the cells. Also, in the case of the post-treatment viability of either the extracts, Zn(II)-Cit, or the mixture thereof, there is marginal to no antioxidant capability under the currently employed conditions. Finally, in the case of V(IV), pre-treatment with either the hybrid material or the mixture of the extracts with the supplementation shows no antioxidant activity, while post-treatment with both the hybrid material and the mixture reveals marginal antioxidant activity.

#### 2.4.2. ROS Detection

**Figure 6:**
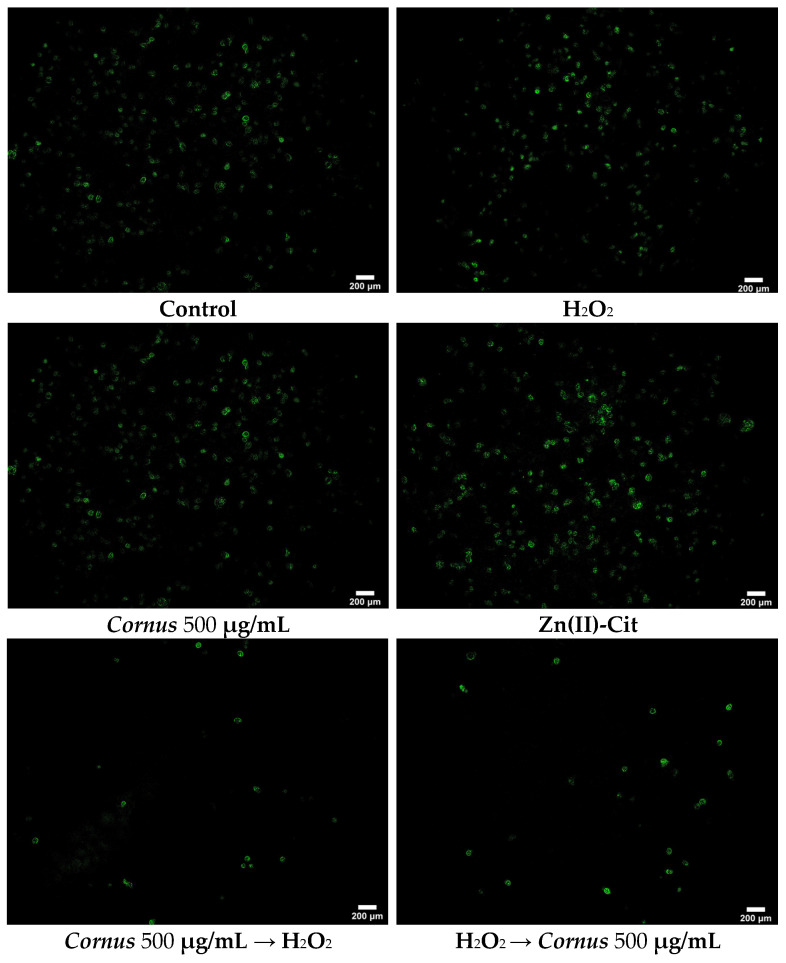

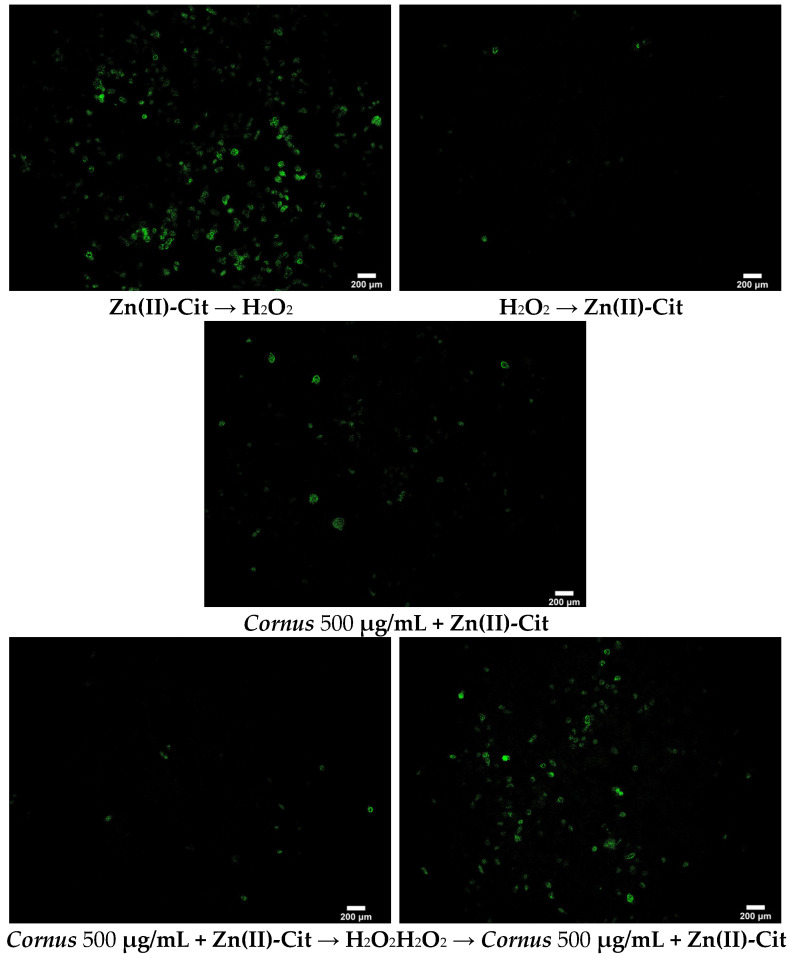
DCF-DA (2′,7′-Dichlorofluorescein diacetate) staining of N2a58 cell cultures, following treatment with *Cornus mas* L. extracts (500 μg/mL), Zn(II)-Cit (100 μM) or the mixture thereof, prior to or after the treatment with H_2_O_2_ (500 μM), along with the corresponding controls.

To further investigate the antioxidant activity of the title materials, DCF-DA staining has been used in N2a58 cell cultures. The results of the visualization of the intracellular ROS emergence are shown in Figure 6. In the case of the H_2_O_2_ treatment, despite the fact that the signal is similar to the control group, more cells were detached from the plate following the treatment, thus leading to a greater signal overall. *Cornus* treatment, either prior to or after the H_2_O_2_ treatment, significantly reduced the ROS signal, thereby leading to a lower signal than the control group. Zn(II)-Cit treatment increases the intracellular ROS, while pre-treatment with the hybrid material seems to have no effect on intracellular stress. At the same time, post-treatment shows a significant reduction in the fluorescent signal. Finally, pre-treatment with the supplemented extracts shows great activity in reducing intracellular oxidative stress, while the post-treatment condition leads to mediocre results.

#### 2.4.3. Antioxidant Gene Expression

**Figure 7:**
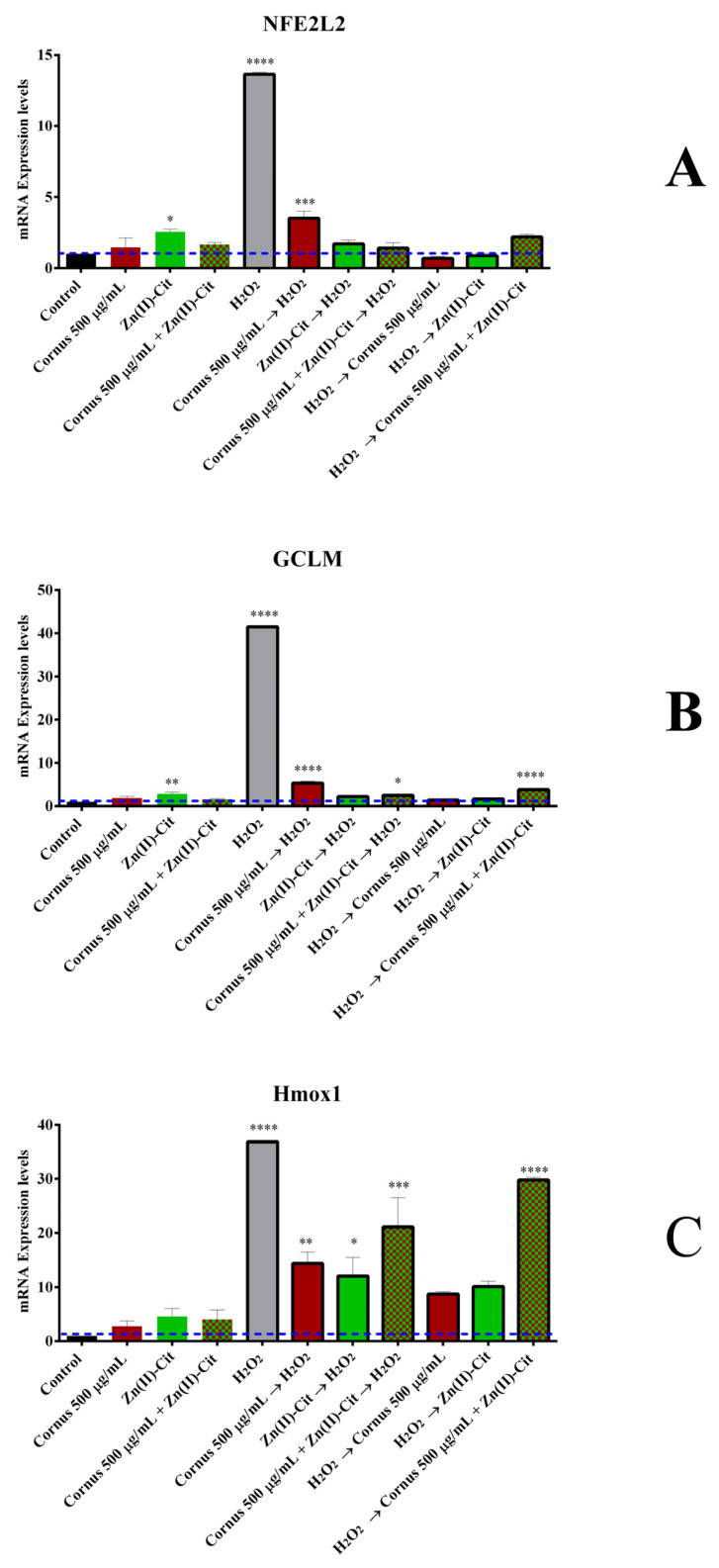
Relative concentration for mRNA expression of (**A**) *NFE2L2*, (**B**) *Hmox1*, and (**C**) *GCLM* in N2a58 cells, following treatment with *Cornus mas* L. extracts (500 μg/mL), Zn(II)-Cit (100 μM) or the mixture thereof, prior to or after treatment with H_2_O_2_ (500 μM), along with the corresponding controls. Significance levels were assessed as follows: * *p* < 0.05 (significant), ** *p* < 0.01 (highly significant), *** *p* < 0.001 (extremely significant) and **** *p* ≤ 0.0001 (extremely significant).

To better understand the mechanism of the antioxidant activity of the materials under investigation, the expression of three different genes was pursued, namely *NFE2L2*, *Hmox1*, and *GCLM*. The results of the mRNA quantification are shown in Figure 7. All three genes follow a similar pattern, where the H_2_O_2_ treatment significantly increases the expression of the genes, and the antioxidant conditions decrease the expression, leaning toward the untreated cell expression levels. Moreover, in all cases, treatment with the antioxidant materials in the absence of the oxidizing agent reveals that the expression of the genes is slightly upregulated. Specifically, in the case of the *NFE2L2* gene, pre-treatment of the extracts alone achieves the highest expression of all antioxidant conditions, while post-treatment with the extracts leads to the lowest expression of all. Likely, from the emerging results, it appears that post-treatment with the extracts shows greater inhibition of the expression of the *NFE2L2* gene compared to the pre-treatment groups (exposure to H_2_O_2_ followed by the addition of the extracts). In the case of *Hmox1*, the results show upregulation of the gene expression under all experimental conditions employed. Post-treatment with zinc-supplemented *Cornus* solution reveals the highest upregulation between the antioxidant conditions, with the lowest one emerging from post-treatment with only *Cornus*. 

Similar to *Hmox1*, *GCLM* gene expression is upregulated in all of the experimental conditions. Post-treatment with both the extracts and Zn(II)-Cit, separately, shows lower upregulation of the gene compared to the pre-treatment group, with the supplemented extracts exhibiting greater upregulation compared to the pre-treated group. The lowest expression between the antioxidant conditions occurs with the post-treatment extracts, projecting a very similar expression to the post-treatment with Zn(II)-Cit, whereas the highest one emerges on pre-treatment with the extracts.

### 2.5. Anti-Inflammatory Studies

**Figure 8:**
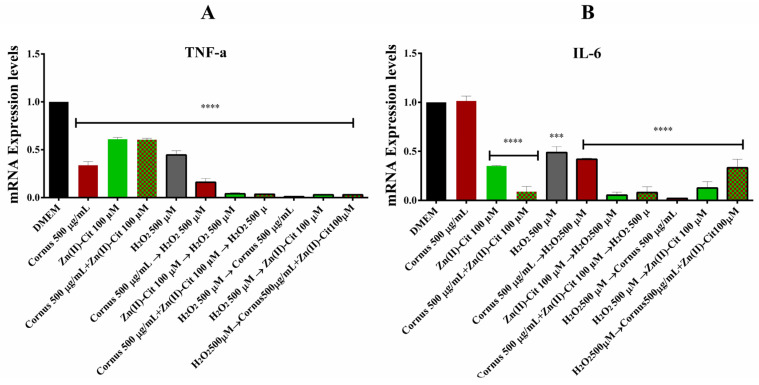
Relative concentration for mRNA expression of (**A**) *TNF-a*; (**B**) *IL-6* in N2a58 cells, following treatment with *Cornus mas* L. extracts (500 μg/mL), Zn(II)-Cit (100 μM) or the mixture thereof, prior to or after the treatment with H_2_O_2_ (500 μM), along with the corresponding controls. Significance levels were assessed as follows: * *p* < 0.05 (significant), ** *p* < 0.01 (highly significant), *** *p* < 0.001 (extremely significant) and **** *p* ≤ 0.0001 (extremely significant).

To further investigate the bioactivity of the extracts, the anti-inflammatory activity of the extracts and the metal complex supplement was evaluated using RT-PCR. The genes studied were *TNF-a* and *IL-6*, two well-known cytokines, involved in the inflammation process. The results of mRNA expression levels, compared to the untreated samples, for the two genes of interest are shown in Figure 8.

In the case of *TNF-a* (Figure 8A), there is downregulation under all experimental conditions tested, with the treatment of the oxidizing agent in the presence of the neuroprotective molecules showing almost complete inhibition of the expression of the gene. By analogy, in the case of the *IL-6* expression (Figure 8B), extract treatment reveals no difference in the expression of the gene, whereas all of the other experimental conditions reveal downregulation of the expression. Moreover, the downregulation of the pre-treatment with the metal complex is greater than that of the extracts alone. In the case of the post-treatment, the opposite pattern is revealed.

### 2.6. Zinc Uptake Studies

**Figure 9:**
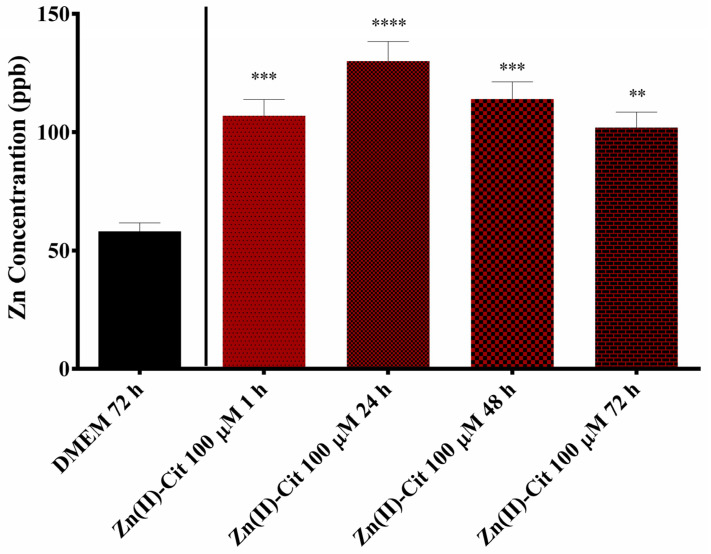
Intracellular concentration of zinc in ppb after treatment with 100 μM of Zn(II)-Cit for 1, 24, 48, and 72 h. Significance levels were assessed as follows: * *p* < 0.05 (significant), ** *p* < 0.01 (highly significant), *** *p* < 0.001 (extremely significant) and **** *p* ≤ 0.0001 (extremely significant).

To further evaluate Zn(II)-Cit as an effective and bioavailable hybrid material, zinc uptake studies were performed in N2a58 cells. For that purpose, the concentration of Zn(II)-Cit and ZnCl_2_ used was 100 μΜ. The results, shown in Figure 9 for Zn(II)-Cit and Figure S14 for ZnCl_2_, reveal, as expected, an increase in intracellular zinc, nearly doubling its concentration inside the cells compared to the untreated cells. Interestingly, the timeframe of the treatment barely affects the intracellular concentration, reaching the maximum concentration at 24 h.

## 3. Discussion

### 3.1. Synthesis–Metal Hybrid Forms

In the quest of providing a highly soluble bioavailable neuroprotective supplementation, natural products have emerged as a logical option for their high biological value, due to the wealth of bioactive compounds in their composition [10]. Specifically, in the case of *Cornus mas* L. extracts, the high concentration of bioactive compounds, such as flavonoids, vitamin C, irridoids, organic acids, and anthocyanins, has emerged as a viable parameter for the pursuit of a soluble nutraceutical extract of high bioactivity [29]. On the other hand, metals are a vital part of human physiology, the deficiencies of which can be attributed to or could contribute to various pathologies [17]. With that in mind, supplementation of the extracts with well-characterized, highly soluble, and bioavailable metal supplements has arisen to further enhance the bioactivity of the extracted biomolecules. In that regard, two appropriately configured and well-defined coordination complexes were employed in this study, Zn(II)-Cit and V(IV)-Cit, in atoxic concentrations, bearing the physiological energy-linked organic substrate of citrate in the coordination sphere of the respective metal ions. The two metal–organic species were fully physicochemically characterized in the solid state and in solution, thus lending credible tools to the investigation of the effects of the *Cornus* extracts on the neuronal cell cultures.

### 3.2. Antioxidant Capacity of Aqueous Cornus Extracts

Preceding the in vitro characterization of the extracts in brain tissue cell cultures, the antioxidant capacity of the extracts was determined spectrophotometrically using the TPC and FRAP assays. The extracts reveal significant antioxidant capacity, comparable to methanolic extraction processes, with the main difference and advantage being the aqueous solubility of the natural product due to the β-cyclodextrin encapsulation process, which provides a carrier with a hydrophilic surface and hydrophobic cavity [30].

### 3.3. Toxicity Profile of Extracts and Hybrid Metalloforms

Prior to the characterization of the bioactivity of the extracts and the metal supplements, the cytotoxicity of all materials was deemed imperative. To provide a model of high biological value to the study, sensitive brain tissue cell lines were employed since the brain is particularly vulnerable to oxidative damage (vide supra). Moreover, to delineate any cytotoxic specificity toward a specific cell line, two different cell lines from different species were used: one human and one mouse. The viability results show atoxicity of the extracts up to very high concentrations (>1 mg/mL), while at the same time, atoxic concentrations of the hybrid materials of Zn(II)-Cit and V(IV)-Cit induce no impairment on the viability of both cell lines. Furthermore, to enhance the atoxic profile of the studied materials, morphological and chemotacticity studies were pursued. In that respect, the observed morphology of the cells was in agreement with the viability results, with the atoxic concentrations employed (C_max_ 100 μΜ in the case of Zn(II)-Cit, and 10 μΜ (N2a58) and 1 μΜ (SH-SY5Y) in the case of V(IV)-Cit) in the viability studies exhibiting normal cell adhesion, cell shape, and cell size, while at concentrations where viability was greatly reduced, cell adhesion was limited, cell size was reduced, and cell shape was distorted. In the case of the motility of the cells, the extracts have a slightly decreasing effect on cell motility, at a concentration where viability is not compromised. On the other hand, the study on the chemotacticity of the metal–organic complex treatment revealed that Zn(II)-Cit has a slight proliferative effect, increasing cell motility, while V(IV)-Cit revealed similar motility with the control. In the case of the metal complex-supplemented extracts, Zn(II)-Cit supplementation increases the motility of the cells, bringing it to comparable levels with that of the Zn(II)-Cit treatment, whereas V(IV)-Cit supplementation reveals no enhancement of the motility of the cells compared to the extracts, thus indicating greater potency of the Zn(II)-Cit treatment in the chemotacticity of the cells.

### 3.4. In Vitro Antioxidant Activity in Cell Cultures

Oxidative stress has been identified as a key factor affecting the pathogenesis and progression of neuronal diseases, including Alzheimer’s disease (AD), Parkinson’s disease (PD), and amyotrophic lateral sclerosis (ALS) [31,32]. Consequently, the antioxidant activity of the potential nutraceutical supplements should be examined. To pursue that, the in vitro antioxidant activity of the extracts and the metal complex supplements was established using viability, ROS staining, and gene expression studies. As a model inducer of oxidative stress, H_2_O_2_, a well-known oxidizing agent, was used. As expected, the oxidizing agent reduces the viability of both cell cultures in all cases, whereas the antioxidant materials behave differently in each case. In the N2a58 cell culture case, pre-treatment with either the extracts or the Zn(II)-Cit supplemented extracts enhances the viability of the cells equally well. On the other hand, pre-treatment only with Zn(II)-Cit shows the greatest increase in cell viability. Moreover, when the antioxidant molecules are added following incubation with the oxidizing agent, the Zn(II)-Cit supplemented experimental condition increases the viability the most. In the SH-SY5Y cell cultures, pre-treatment with the materials reveals similar results as in the N2a58 cell culture, while in the post-treatment case, only the Zn(II)-Cit treatment alone provides partial alleviation from oxidative damage. In the case of V(IV)-Cit, the antioxidant effect is evident only in the case of SH-SY5Y, following induction of oxidative damage. From the viability studies, it is evident that Zn(II)-Cit exhibits better antioxidant activity than V(IV)-Cit, enhancing the antioxidant properties of the extracts, while at the same time, the effect is more evident in N2a58 than in SH-SY5Y cell cultures (cell tissue-specific behavior). For these reasons, ROS staining and mechanistic studies have been performed in N2a58 cell cultures. The antioxidant properties of the extracts with and without supplementation of Zn(II)-Cit were investigated using DCF-DA staining of ROS. The visualization of ROS provides similar results with the viability assessment, further enhancing the antioxidant profile of the extracts and the supplement. Finally, to complete the antioxidant profile of the studied conditions, the expression levels of three genes implicated in the antioxidant response of the cells were quantified. Specifically, the three genes that were studied included *NFE2L2*, *Hmox1*, and *GCLM*. These three genes are implicated in the Keap1–Nrf2 antioxidant pathway. Specifically, the Nrf2 antioxidant transcription factor (expressed through the *NFE2L2* gene) is bound to the Keap1 protein in the cytosol, under basal homeostatic conditions. However, under oxidative stress conditions, Nrf2 dissociates from Keap1 and translocates inside the nucleus of the cell, where it binds to the ARE (Antioxidant Response Elements) region, thereby expressing specific cytoprotective target genes, responsible for detoxification, antioxidation, and metabolism. Two such genes, regulated by the Nrf2 transcription factor, are *Hmox1* and *GCLM* [33,34,35,36]. From the present study, it is evident that in all three genes, treatment with the extracts, Zn(II)-Cit or a combination thereof, slightly upregulates the expression of the genes, while the oxidizing agent upregulates significantly more the expression of these genes. Subsequently, pre-treatment or post-treatment of the antioxidant molecules, in the presence of the oxidizing agent, reduces the expression of the genes toward the expression levels of the control, thus verifying the above-mentioned antioxidant properties and concurrently providing evidence regarding the mechanism of action of the materials.

### 3.5. Anti-Inflammatory Activity in Cell Cultures

Neuroinflammation refers to the immune response of the central nervous system (CNS) to harmful stimuli, with the primary function to restore CNS homeostasis and repair the damage. However, chronic or dysregulated neuroinflammation can lead to the onset of neurodegeneration. Actually, chronic neuroinflammation has been recognized as a core feature of neurodegenerative diseases, with persistent glial cell activation leading to the continuous release of proinflammatory cytokines, chemokines, and reactive oxygen species (ROS), which contribute to the accumulation of neurotoxic proteins, promoting neuronal damage [37]. Inflammatory mediators play a crucial role in the initiation, amplification, and resolution of the inflammatory response. The activation of the macrophages releases a variety of inflammatory mediators, such as cytokines, chemokines, and lipid mediators. The most typical proinflammatory mediators include IL-1β, TNF-α, IL-6, and iNOS, while release of mediators, such as IL-4, IL-10, and neurotrophic factors, signals the anti-inflammatory phenotype [38,39]. TNF-α and IL-6 are typical multifunctional proinflammatory cytokines, with widely overlapping biological functions, yet they both show distinct characteristics, through which they were discovered, namely IL-6 for its antibody production, with TNF-α as an endotoxin-induced factor causing tumor necrosis. At the same time, it must be noted that these cytokines interact with each other, regulating their expression patterns [40]. In this study, *TNF-a* and *IL-6* expression levels were evaluated, and the expression pattern reveals significant downregulation of the expression of both genes, thus validating the anti-inflammatory action of the extracts and the Zn(II)-Cit supplement.

### 3.6. Zn-Uptake Studies

Finally, the bioavailability of the Zn(II)-Cit supplement was evaluated using zinc uptake studies in N2a58 cell cultures and the results were compared to ZnCl_2_. Juxtaposed to the untreated sample, both Zn(II)-Cit and ZnCl_2_ treatments increased the intracellular concentration of zinc, with both concentrations peaking at 24 h treatments. At the same time, the Zn(II)-Cit treatment increase was marginally greater in each timeframe tested, with the maximum intracellular concentration reaching 130 ± 12 ppb achieved in a 24 h treatment, compared to 125 ± 11 ppb for ZnCl_2_, thus indicating that the hybrid metal–organic compound could be further used as competitive metal source compared to metal salts [41] in view of the advantages emanating from the source of the metal-bound energy-linked organic tricarboxylic acid substrate (vide infra). 

## 4. Materials and Methods

### 4.1. Reagents

For the synthesis of the hybrid materials, ZnCl_2_ was purchased from Merck (Darmstadt, Germany), anhydrous citric acid from ChemLab (Zedelgem, Belgium), ammonia from VWR Chemicals (Darmstadt, Germany), VCl_3_ from Aldrich (Waltham, MA, USA), and sodium hydroxide from Sigma-Aldrich (St. Louis, MO, USA).

For the photometric evaluation of the total phenolic contents (TPC) and Ferric Reduction Antioxidant Power (FRAP) of the extracts, the Folin–Ciocalteau (F–C) reagent was purchased from Supelco-Sigma (Steinheim, Germany), sodium carbonate from P. Bacacos (Thessaloniki, Greece), gallic acid from Sigma (Steinheim, Germany), ascorbic acid from BDH Chemicals (Darmstadt, Germany), acetic acid from Aldrich (Waltham, MA, USA), sodium acetate from Johnson Mathey (London, UK), 2,4,6-Tri(2-pyridyl)-1,3,5-triazine (TPTZ) from Alfa Aesar (Ward Hill, MA, USA), hydrochloric acid was obtained from Ferak (Berlin, Germany), iron(III) chloride was purchased from Fluka (Gillingham, UK), and ultrapure water was produced by our laboratory.

For the in vitro studies, High Glucose Dulbecco’s Modified Eagle’s Medium (DMEM) with stable Glutamine and sodium pyruvate (L-103), Dulbecco’s Modified Eagle’s Medium supplemented with Ham’s F-12 Nutrient Mixture in a volumetric ratio of 1:1 (DMEM-F12) with stable Glutamine and 15 mM Hepes (L0092), Fetal Bovine Serum (FBS) South America origin (S1810), penicillin–streptomycin (P/S) solution 100× (L0022), and trypsin-EDTA 1X solution in Phosphate Buffer Saline (PBS) w/o calcium, w/o magnesium and w/o phenol red (L0940) were purchased from Biowest (Nuaillé, France), with trypan blue purchased from Sigma (Steinheim, Germany). PBS (1× pH 7.4) was prepared by dissolving sodium chloride (Panreac AppliChem, Barcelona, Spain), disodium phosphate (Merck, Darmstadt, Germany), potassium dihydrogen phosphate (Panreac AppliChem, Spain) and potassium chloride (Riedel-de Haën, Hannover, Germany) under continuous stirring in sterile ultrapure water in our laboratory. The resulting solution was buffered using HCl (Ferak, Berlin, Germany) 1 M solution to pH 7.4 and then additional sterile ultrapure water was added. Subsequently, the solution was autoclaved and filtered prior to use. The XTT (2,3-bis-(2-methoxy-4-nitro-5-sulfophenyl)-2H-tetrazolium-5-carboxanilide) cell viability kit was purchased from Cell Signaling Technologies (Danvers, MA, USA), Triton X-100 for Molecular Biology from Sigma (Steinheim, Germany), hydrogen peroxide (H_2_O_2_) from ChemLab (Zedelgem, Belgium), 2′,7′-dichlorofluorescin diacetate (DCF-DA) from Sigma (Steinheim, Germany), paraformaldehyde (PFA) from BDH Chemicals (Darmstadt, Germany), water for molecular biology and Tritidy G were purchased from Panreac AppliChem (Spain), chloroform was purchased from VWR Chemicals (Darmstadt, Germany), ethanol from J.T. Baker (Phillipsburg, NJ, USA), isopropanol from Sigma (Steinheim, Germany), nitric acid from ChemLab (Zedelgem, Belgium), EDTA (Ethylenediaminetetraacetic acid) from Panreac (AppliChem, Spain), and RNase-free water from Qiagen (Hilden, Germany). All of the primers were pre-designed LNA (Locked Nucleic Acid) primers for each gene purchased from Qiagen (Hilden, Germany), with the cDNA synthesis kit (iScript gDNA Clear cDNA Synthesis Kit, 1725035) and PCR master mix (iTaq Universal SYBR Green Supermix, 1725120) having been purchased from Bio-Rad (Hercules, CA, USA).

### 4.2. Synthesis of Metal–Organic Hybrid Complex Materials

#### 4.2.1. Synthesis of Zinc Citrate (Zn(II)-Cit) 

Zinc citrate (Zn(II)-Cit) was synthesized as described in the literature, with slight modifications [42]. Briefly, zinc chloride (ZnCl_2_) and citric acid, in a molar ratio of 1:10, were dissolved in water, and their pH was adjusted to ~7.5, using aqueous ammonia. Ammonia acts both as a base to increase the pH of the solution while it concurrently provides the counter ions to the emerging anionic complex in (NH_4_)_4_[Zn(C_6_H_5_O_7_)_2_] (Zn(II)-Cit). The crystalline hybrid material was characterized through elemental analysis, spectroscopically, using FT-IR (Nicolet IR200 Infrared Spectrometer, Thermo-Fischer Scientific, Madison, WI, USA), and crystallographically, using a Bruker Kappa APEX II (Bruker, Bremen, Germany) diffractometer equipped with a triumph monochromator and Mo *K*α radiation.

#### 4.2.2. Synthesis of Vanadium Citrate (V(IV)-Cit) 

Vanadium citrate Na_4_[V_2_O_2_(C_6_H_4_O_7_)_2_]•12H_2_O (V(IV)-Cit) was produced and isolated as crystalline material using the methodology described in the literature, with slight modifications [43]. In brief, vanadium(III) chloride and citric acid were mixed in water in a molar ratio of 1:1, the solution pH was adjusted to 8, using aqueous sodium hydroxide solution, and the reaction mixture was left for overnight stirring. On the following day, the blue solution was dried in vacuo, the residue was redissolved in water, and crystalline material was isolated a few days later using isopropanol as a precipitating agent. The sodium hydroxide solution, apart from the increase in pH, acts also as the source of counter ions to the anionic complex [V_2_O_2_(C_6_H_4_O_7_)_2_]^4−^. The hybrid material was characterized through elemental analysis, spectroscopically, using the FT-IR technique (Nicolet IR-200 Infrared Spectrometer, Thermo-Fischer Scientific, Madison, WI, USA), and crystallographically, using Bruker Kappa APEX II (Bruker, Germany) diffractometer equipped with a triumph monochromator and Mo *K*α radiation.

#### 4.2.3. Extraction and Isolation of *Cornus mas* L. Extracts

The lyophilized extracts were produced and optimized as described in the literature [26]. Briefly, after washing and destoning a batch of fresh Cornelian cherries, their juice was removed, and the produced pomace was freeze-dried until further use. The extraction of the bioactive compounds was optimized using ultrasound-assisted extraction with independent variables: liquid-to-solid ratio (L/S), β-cyclodextrin concentration (Cβ-cd), in the aqueous solution, and sonication amplitude and duration, with the dependent responses being the total phenolic content (TPC), total anthocyanin content (TMA), DPPH radical scavenging activity, and loganic acid content. The optimized conditions were L/S of 50 mL/g, C_β-cd_ was 4.5 mg/mL, sonication amplitude 90%, and sonication time 22 min. The following studies were performed using optimized extracts, which were lyophilized and kept at −20 °C until further use. The desired solutions for the study of the extracts were freshly prepared prior to the experiments.

### 4.3. Determination of Total Phenolic Content (TPC) and Ferric Reduction Antioxidant Power (FRAP)

The photometric antioxidant capacity of aqueous *Cornus* extracts was investigated using the total phenolic content (TPC) [44] and Ferric Reduction Antioxidant Power (FRAP) [45] assays, as reported in the literature, with slight modifications. Specifically, for TPC determination, 2.8 mL of ultrapure water was mixed with 0.1 mL aqueous extract solution and 0.1 mL F–C reagent. The emerging yellow mixture was vortexed for 1 min. To that reaction mixture, 2 mL of aqueous sodium carbonate solution 7.5% *w/v* was added, and the blue solution was kept in the dark for 1 h. The absorbance of the final mixture was measured at 750 nm on a Hitachi U-1900 spectrophotometer (Hitachi, Ibaraki, Japan), and the results were expressed as mg GAE/g dry extract, using a gallic acid (68 mg/L–510 mg/L) calibration curve (y = 300.437x + 0.0034, r^2^ = 0.998).

For determination of the ability of the extracts to reduce ferric ions (FRAP assay), the following solutions were prepared: 300 mM acetate buffer (CH_3_COOH/CH_3_COONa, with the pH adjusted to 3.6 using HCl (1 M) or NaOH (1 M)); 10 mM TPTZ prepared in 40 mM HCl; 20 mM FeCl_3_ 6H_2_O solution in ultrapure water. The FRAP reagent was prepared by appropriate mixing of the aforementioned solution acetate buffer:TPTZ:iron chloride in a ratio 10:1:1. A volume of 80 μL of the desired aqueous *Cornus* extracts was mixed with 4 mL of the FRAP reagent, and the mixture was left for 15 min at 37 °C in the dark. Subsequently, the absorbance of the navy-blue solution was measured at 596 nm on a Hitachi U-1900 spectrophotometer (Hitachi, Ibaraki, Japan). The results are expressed in mg AAE/g dry extract, using an ascorbic acid (124 mg/L–1000 mg/L) standard calibration curve (y = 988.219x + 0.029, r^2^ = 0.999). Five replicates of each assay were run and the results are expressed as mean ± standard deviations (SD).

### 4.4. Cell Lines

For the in vitro evaluation of the potency of the extracts, two sensitive neuroblastoma cell lines were used, i.e., N2a58 (murine) and SH-SY5Y (human). The N2a58 cells were cultured in DMEM media, whereas the SH-SY5Y cells were cultured in DMEM-F12 media. Both media were supplemented with a 1% *v*/*v* penicillin-streptomycin solution and 10% *v/v* FBS to generate the complete media, while both cell lines were cultured in a humidified incubator at 37 °C and 5% CO_2_. When appropriate confluency was achieved, the medium of the cells was removed, and the attached cells were rinsed with PBS. To detach the cells, trypsin was added, and the plate was incubated at 37 °C. Subsequently, a complete medium was added to inactivate trypsin and the resulting mixture was centrifuged. The cell pellet was resuspended in a complete medium, and the concentration of viable cells was evaluated using the trypan blue exclusion assay.

### 4.5. Cytotoxicity Evaluation

To evaluate the cytotoxicity profile of the extracts, the supplementary hybrid compounds Zn(II)-Cit and V(IV)-Cit, as well as combinations thereof, apart from cell viability, we studied the morphology and chemotacticity of the cells in each case, in a concentration-, time-, and cell line-dependent fashion.

#### 4.5.1. Viability Evaluation

For the viability evaluation of the extracts and the hybrid compounds Zn(II)-Cit and V(IV)-Cit, as well as combinations thereof, the XTT cell viability assay was used according to manufacturer instructions. To pursue that, 5000 cells in 100 μL of the proper medium were seeded per well of a tissue culture 96-well plate, and the plate was left overnight in the incubator for the cells to attach to the plate surface. On the following day, the medium was replaced by 100 μL of medium, containing the title compound concentration, and the plate was incubated for the designated time. At the end of the timeframe, 50 μL of XTT detection solution (containing 49 μL of XTT reagent and 1 μL of electron coupling solution) was added in each well and the plate was incubated for 4 h. Subsequently, the absorbance A of each well was measured at 450 nm, using a BioTek Synergy H1 Multimode Reader (Agilent Technologies, Santa Clara, CA, USA). The survival percentage was calculated by subtracting the absorbance of the culture medium (CM) without cells from the absorbance of each experimental condition (EC), and subsequently dividing this difference with the difference in absorbance of the control medium with cells minus the absorbance of the control medium without cells, and multiplying with 100, as shown below. All of the experiments were performed in triplicates.

$$\%Viability=100\times \frac{A_{450(EC)}-A_{450\left(CM\;no\;cells\right)}}{A_{450\left(CM\;with\;cells\right)}-A_{450\left(CM\;no\;cells\right)}}$$

#### 4.5.2. Morphology Studies

To better evaluate the cytotoxicity profile of each of the experimental conditions, morphology studies were pursued. To that end, 200,000 cells in 2 mL of proper medium were seeded per well of a tissue culture six-well plate, and the plate was incubated overnight. On the following day, the medium was replaced by 2 mL of medium containing the title concentration in each case. At specific time points (0, 24, 48, and 72 h), the plate was photographed via an Oxion Inverso biological microscope (Euromex, Duiven, The Netherlands) using both 10× and 40× lenses.

#### 4.5.3. Chemotacticity

The chemotacticity of the cells under the specific experimental conditions was evaluated using the wound-healing (scratch) assay. Briefly, 200,000 cells in 2 mL of proper medium were seeded per well of a tissue culture six-well plate, and the plate was incubated until confluency had reached 80–90%. Subsequently, with a sterile yellow 200 μL tip, a vertical line was drawn across the well, thus disassociating the attached cells from the plate. Then, the medium of the well was replaced by 2 mL of medium containing the title compound concentration, and pictures were taken at specific time points (0, 24, 48, and 72 h) (vide supra) using the 10× lens. The pictures were imported in ImageJ software (version 1.53t, NIH, Bethesda, MD, USA), and the scratch distance was measured at each time point.

### 4.6. Antioxidant Activity

To evaluate the antioxidant potency of the extracts and the effectiveness of the supplementation using the hybrid compounds Zn(II)-Cit and V(IV)-Cit, as well as combinations thereof, H_2_O_2_, a well-known oxidant, was used to induce oxidative stress. For more information regarding the antioxidant properties, apart from the evaluation of the viability under oxidative stress, reactive oxygen species (ROS) were visualized using DCF-DA staining, and the cellular mechanism was studied using RT-PCR (Reverse Transcriptase Polymerase Chain Reaction).

#### 4.6.1. Viability Under Oxidative Stress

To evaluate the viability of the cell cultures under oxidative stress and the potency of the extracts and Zn(II)-Cit to increase viability, the XTT viability assay was used. The protocol was similar to the one mentioned above, but after overnight incubation, the medium was replaced by one containing H_2_O_2_. After 2 h of incubation with the oxidizing agent, the antioxidant agent (extracts, Zn(II)-Cit, combination thereof) was added, and the plate was incubated for an additional 24 h. Subsequently, the XTT assay was performed as mentioned above. Moreover, experiments were performed where the antioxidant was added prior to the oxidizing agent (2 h antioxidant pre-treatment). All of the experiments were performed in triplicates.

#### 4.6.2. DCF-DA (2′,7′-Dichlorofluorescein Diacetate) Staining

To visualize the reduction of ROS in the presence of our potential antioxidant agent(s), the DCF-DA staining protocol was performed. Briefly, 200,000 N2a58 cells in 2 mL were seeded in small tissue culture dishes (35 × 10 mm) and incubated overnight. The medium was replaced by 2 mL of medium containing the title compound similar to the XTT assay (antioxidant treatment both prior to and after induction of the oxidative stress). After the end of the chosen timeframe, the treatment solution was removed, and the cells were rinsed twice with 2 mL PBS. After that, 1.5 mL of 4% PFA in PBS was added and the dishes were incubated for 30 min at room temperature. Subsequently, the PFA solution was discarded, cells were rinsed twice with 2 mL PBS, and 1.5 mL of 1 μM DCF-DA dye in PBS was added. The dishes were incubated for 30 min at 37 °C, and then the dye solution was discarded. Finally, after a rinse with PBS, 2 mL of PBS was added and the dishes were visualized using fluorescence microscope Axio Observer Z1 (Carl Zeiss, GmbH Jena, Jena, Germany). Images were captured on an AxioCam Hc camera (Carl Zeiss, GmbH Jena, Germany) and processed using ImageJ software (version 1.53t, NIH, Bethesda, MD, USA).

#### 4.6.3. RT-PCR (Reverse Transcriptase Polymerase Chain Reaction) Assay 

To evaluate the mechanism of action triggered by the presence of oxidizing and antioxidant agents, RT-PCR was used. Briefly, 200,000 N2a58 cells were seeded per well of a tissue culture six-well plate and incubated overnight. After the desired treatment with the oxidizing and/or the antioxidant agents (vide supra), treatment solutions were discarded, and cells were lysed using 1 mL of Tritidy G per well. The plate was subsequently incubated at room temperature for 20 min in the dark. The lysate was transferred to an Eppendorf tube, 200 μL of chloroform was added, and after shaking, the reaction mixture was incubated at room temperature for 15 min. After centrifugation at 12,000× *g* for 15 min at 4 °C, the upper clear phase (containing the RNA) was isolated, and 0.5 mL of ice-cold isopropanol was added. The resulting mixture was incubated at −20 °C for 45 min. The Eppendorf tube was centrifuged (vide supra), and the pellet was isolated and washed consecutively with 1 mL of 75% ethanol in sterile ultrapure water and then with 1 mL of pure ethanol. After the last wash, the supernatant was discarded, and the pellet was air-dried prior to resuspension of the RNA pellet in RNase-free water. The purity and concentration of the extracted RNA were evaluated using Nanodrop 2000 (Thermo Scientific, Waltham, MA, USA). Subsequently, 1 μg of RNA was used to produce cDNA, using the cDNA synthesis kit (iScript gDNA Clear cDNA Synthesis Kit) (Bio-Rad, Hercules, CA, USA) according to the manufacturer’s instructions. The cDNA concentration was determined using Nanodrop 2000 (Thermo Scientific, Waltham, MA, USA). PCR amplification was performed in a Rotor-Gene Q (Qiagen, Hilden, Germany) thermocycler, using mouse LNA primers for the genes: *GAPDH* (MM_GAPDH_2176592), *NFE2L2* (MM_NFE2L2_2096657), *GCLM* (MM_GCLM_2188527), and *Hmox1* (MM_HMOX1_2180547), with the following protocol: 95 °C 10 min, 40 cycles of 95 °C for 15 s, 60 °C for 30 s, and 72 °C for 30 s, followed by a hold at 72 °C for 5 min. The fluorescent signal was detected in the channel for the SYBR Green dye, and the results were quantified using delta delta Ct (2^−ΔΔCt^) analysis compared to the untreated condition, with *GAPDH* serving as the housekeeping gene.

### 4.7. Anti-Inflammatory Activity

To evaluate the anti-inflammatory activity of the extracts and the effect(s) of Zn(II)-Cit supplementation, RT-PCR was used. The treatment of the cells, the RNA extraction, and the cDNA synthesis were similar to the aforementioned protocol. For the primers, mouse LNA primers were used for the genes *GAPDH* (vide supra), *TNF-a* (MM_TNF_2032539), and *IL-6* (MM_IL6_1986720), with the following protocol: 95 °C 2 min and 50 cycles of 95 °C for 5 s,60 °C for 10 s. The fluorescent signal was detected in the channel for the SYBR Green dye, and the results were quantified using delta delta Ct (2^−ΔΔCt^) analysis, compared to the untreated condition, with *GAPDH* being the housekeeping gene.

### 4.8. Metal Uptake Studies

To further delineate the effects of hybrid Zn(II)-Cit material in the cells, zinc uptake studies were performed in N2a58 cell cultures. Briefly, after the seeding of 200,000 N2a58 cells and their overnight incubation at 37 °C and 5% CO_2_, cells were treated with 100 μM of Zn(II)-Cit for 1, 24, 48, and 72 h. All of the tips and the Eppendorf tubes used in this study were incubated overnight in 5% nitric acid solution and then rinsed with ultrapure water. Following the treatment of the cells, the solution was removed and kept in an Eppendorf tube; the cell monolayer was rinsed with 1 mL of 1 mM EDTA solution in PBS, a well-known and very potent zinc chelator [46], to remove any loosely bound zinc residues from the cells. Subsequently, the cells were rinsed with PBS. To disassociate the cell monolayer from the plate, 1.5 mL of trypsin solution was added, and incubation took place at 37 °C for 5 min. To make sure that all of the cells had been disassociated from the plate, following trypsin incubation, the solution was pipetted several times and then stored in an Eppendorf tube. The samples were frozen immediately at −20 °C until the time of the measurement. Inductively Coupled Plasma Mass Spectrometry (ICP-MS) analysis was carried out following established protocols described in the literature [47], utilizing an Agilent 7500CE ICP-MS instrument (Agilent Technologies, Santa Clara, CA, USA), a high sensitivity and precise multielement detection instrumentation. Sample preparation was conducted using a CEM MARS 6 microwave digestion system (CEM Corporation, Matthews, NC, USA) to efficiently break down complex matrices for trace elemental analysis. The digestion procedure involves treating the samples with an acid mixture composed of concentrated nitric acid (HNO_3_) and hydrogen peroxide (H_2_O_2_) in a volumetric ratio of 4:1. This mixture facilitates oxidative degradation of organic material and solubilization of metals. The digestion was performed under controlled conditions of elevated pressure, maintained at 200 psi, with a rise to pressure ramp followed by a 10-minute hold time to ensure complete sample dissolution and consistent results. These stringent parameters enhance the complete digestion of the samples investigated. Following the digestion, the samples are introduced into the Inductively Coupled Plasma Mass Spectrometry (ICP-MS) instrument (Agilent Technologies, Santa Clara, CA, USA), where a high-temperature plasma (approximately 10,000 K) ionizes the elements within the sample. The resulting ions are separated based on their mass-to-charge ratios using a quadrupole mass analyzer, enabling accurate quantification of trace elements. The intracellular concentrations were expressed as ppb (parts per billion). In the case of V(IV)-Cit, similar studies were not performed, mainly due to the lack of antioxidant activity (vide supra) and the low concentration of the treatment solution.

### 4.9. Statistical Analysis

The obtained experimental data are presented as average ± standard error mean (SEM) values of multiple sets of three independent measurements for the in vitro results and as average ± standard deviation (SD) values of multiple sets of independent measurements for the analytical studies. Mean cell survival rates and SEMs were calculated for each individual group. *p*-values were calculated using either one- or two-way analysis of variance, followed by Dunnet’s method, using GraphPad Prism v.6. Significance levels were assessed as follows: * *p* < 0.05 (significant), ** *p* < 0.01 (highly significant), *** *p* < 0.001 (extremely significant) and **** *p* ≤ 0.0001 (extremely significant) or non-significant (*p* > 0.05).

## 5. Conclusions

The pursuit of potent nutraceutical supplements counteracting the effects of oxidative stress linked to neurodegeneration has led to the utilization of bioactive materials derived from natural products to ensure their low cytotoxicity. Therefore, the employed approach targeted the use of natural extracts from *Cornus mas* L. and well-defined hybrid meta-organic complex species comprised of physiologically relevant constituents exemplified in Zn(II)-Cit and V(IV)-Cit, individually and collectively investigating potency, activity and potential synergism between the emerging binary bioactive combinations. To that end, the study focused and evaluated the bioactive profile of optimized aqueous extracts of *Cornus mas* L. photometrically, using FRAP and TPC methods, and determined in vitro their multifaceted activity, using two sensitive brain tissue cell lines; one human (SH-SY5Y) and one mouse (N2a58). To that end, the TPC and FRAP assays emphasized the significant antioxidant capacity of the aqueous extracts, thus justifying further work toward in vitro evaluation. The in vitro evaluation of the extracts provided the cytotoxicity profile in a dose-, time- and cell tissue-dependent fashion, evaluating not only the viability of cells but also the morphology and chemotacticity in each case, always factoring in the contribution of the zinc and vanadium complex species. The complete profile of cytotoxicity, evaluating the viability, morphological, and chemotactic data, indicates that the extracts are not toxic up to very high concentrations, while at the same time, supplementation of the extracts with the maximum atoxic concentrations of the hybrid materials revealed no additional cytotoxicity. Moreover, the extracts exhibit significant antioxidant activity, which was assessed through viability, ROS staining, and PCR studies, further enhanced by Zn(II)-Cit supplementation against H_2_O_2_-induced oxidative stress. Concurrently, the downregulation of pro-inflammatory genes (*IL-6* and *TNF-α*) indicated the anti-inflammatory activity of the extracts, enhanced by Zn(II)-Cit supplementation. Finally, bioavailability studies on Zn(II)-Cit show that there are new potentially competitive metal–organic compounds of metal salt sources of Zn(II), exhibiting (a) solubility and biochemical reactivity characteristics commensurate with the nature and role of the organic acid in energy produced through cellular physiology, and (b) significant antioxidant activity commensurate with the nature of the organic acid substrate and the hybrid nature of a promising neuroprotective agent. These results provide solid evidence that zinc supplementation under the employed experimental conditions has a significant neuroprotective effect, with no ostensible undesired effects subverting neuronal physiology and integrity. The collectively formulated bioactivity profile of the extracts and the employed metal–organic species provide solid evidence of the tissue cell-specific beneficial nutraceutical properties of the *Cornus mas* L. extracts, further enhanced by a well-defined soluble and bioavailable hybrid form of zinc, thus setting the groundwork for further assessment of such composite natural products in averting oxidative stress-induced neurodegenerative events in sensitive human tissue loci. In pursuit of such a goal, the in vivo bioactivity profile of the above-mentioned materials needs to be investigated, in order to (a) validate the effectiveness of the extracts with and without the supplements, and (b) delineate the role of each participant in the formulation of new neutraceuticals acting proactively to protect, avert, and/or retard neurodegeneration.

## Figures and Tables

**Table 1:** TPC and FRAP content of *Cornus mas* L. extracts.

	TPC (mg GAE/g Dry Extracts)		FRAP (mg AAE/g Dry Extracts)
	6.46		9.56
	6.37		9.62
	6.49		9.53
	6.64		9.52
	6.43		9.49
Mean Value	6.48	Mean Value	9.54
Std. Deviation	0.09	Std. Deviation	0.04

**Table 2:** Migration speed of SH-SY5Y cells in the presence of extracts, hybrid materials, and a combination of both.

Concentration		Migration Speed (mm/h)
Control		0.00947 ± 0.00100
Extracts	100 μg/mL	0.00719 ± 0.00200
	1 mg/mL	0.00735 ± 0.00100
Zn(II)-Cit	100 μM	0.0120 ± 0.0010
V(IV)-Cit	500 nM	0.00970 ± 0.00200
Extracts + Zn(II)-Cit		0.0119 ± 0.0010
Extracts + V(IV)-Cit		0.00712 ± 0.00100

## Data Availability

Data are contained within the article and Supplementary Materials.

## Supplementary Materials

The following supporting information can be downloaded at: https://www.mdpi.com/article/10.3390/ijms26031159/s1.

## Abbreviations

ANOVA	Analysis of Variance
C_β-cd_	β-Cyclodextrin concentration
c-3-g	Cyanidin-3-glucoside
DCF-DA	2′,7′-Dichlorofluorescein diacetate
DPPH	2,2-Diphenyl-1-picrylhydrazyl
DMEM	Dulbecco’s modified Eagle’s medium
DMEM-F12	Dulbecco’s Modified Eagle’s Medium supplemented with Ham’s F-12 Nutrient Mixture (1:1 *v*/*v*)
EDTA	Ethylenediaminetetraacetic acid
FBS	Fetal Bovine Serum
FRAP	Ferric Reduction Antioxidant Power
F–C reagent	Folin–Ciocalteau reagent
GAE	Gallic acid equivalents
GAPDH	Glyceraldehyde 3-Phosphate Dehydrogenase
GCLM	Glutamate-Cysteine Ligase Modifier Subunit
Hmox1	Heme Oxygenase 1
IL-1β	Interleukin 1 beta
IL-6	Interleukin 6
L/S	Liquid to solid ratio
NFE2L2	Nuclear Factor Erythroid-derived 2-like 2
PBS	Phosphate Buffer Saline
PFA	Paraformaldehyde
ppb	Parts per billion
ROS	Reactive Oxygen Species
RT-PCR	Reverse Transcriptase Polymerase Chain Reaction
SD	Standard deviation
SEM	Standard error mean
TNF-α	Tumor Necrosis Factor alpha
TPC	Total Phenolic Content
TPTZ	2,4,6-Tri(2-pyridyl)-1,3,5-triazine
V(IV)-Cit	Vanadium(IV) Citrate, Na_4_[V_2_O_2_(C_6_H_4_O_7_)_2_]·12H_2_O
XTT	(2,3-bis-(2-methoxy-4-nitro-5-sulfophenyl)-2H-tetrazolium-5-carboxanilide)
Zn(II)-Cit	Zinc Citrate, (NH_4_)_4_[Zn(C_6_H_5_O_7_)_2_]
