# Supplementary material for: Cornus mas L. Extracts Exhibit Neuroprotective Properties, Further Enhanced by Metal-Bound Energy-Linked Organic Substrates
Source: Int J Mol Sci. 2025 Jan 29;26(3):1159. doi: 10.3390/ijms26031159 (PMC11817807; doi:10.3390/ijms26031159)
Supplement: Supplementary file 1 [file ijms-26-01159-s001.zip › ijms-3421671-supplementary.pdf]

SUPPLEMENTARY MATERIAL

Cornus mas L. extracts exhibit neuroprotective properties, further enhanced by metal-bound energy-linked organic substrates

Georgios Lazopoulos,[a] Sevasti Matsia,[a] Marios Maroulis,[a] Athanasios Salifoglou,[a]*

* Author to whom correspondence should be addressed.
Tel: +30-2310-996-179, E-mail: **salif@auth.gr**

[a] Laboratory of Inorganic Chemistry and Advanced Materials, School of Chemical Engineering, Aristotle University of Thessaloniki, Thessaloniki 54124, Greece

Figure captions

Figure S1: **A.** Survival percentage of N2a58 cells, after treatment with various concentrations (10 pg/mL to 10 mg/mL) of *Cornus mas* L. extracts, for 24, 48 and 72 h, compared to the untreated samples (control).

B. Survival percentage of SH-SY5Y cells, after treatment with various concentrations (10 pg/mL to 10 mg/mL) of *Cornus mas* L. extracts, for 24, 48 and 72 h, compared to the untreated samples (control). Significance levels were assessed as follows: * $p < 0.05$ (significant), ** $p < 0.01$ (highly significant), *** $p < 0.001$ (extremely significant) and **** $p \leq 0.0001$ (extremely significant).

Figure S2: **A.** Survival percentage of N2a58 cells, after treatment with various concentrations (1 nM to 1 mM) of Zn(II)-Cit hybrid material, for 24, 48 and 72 h, compared to the untreated samples (control).

B. Survival percentage of SH-SY5Y cells, after treatment with various concentrations (1nM to 1mM) of Zn(II)-Cit hybrid material, for 24, 48 and 72 h, compared to the untreated samples (control). Significance levels were assessed as follows: * $p < 0.05$ (significant), ** $p < 0.01$ (highly significant), *** $p < 0.001$ (extremely significant) and **** $p \leq 0.0001$ (extremely significant).

Figure S3: **A.** Survival percentage of N2a58 cells, after treatment with various concentrations (1 μM to 100 μM) of V(IV)-Cit hybrid material, for 24, 48 and 72 h, compared to the untreated samples (control).

B. Survival percentage of SH-SY5Y cells, after treatment with various concentrations (500nM to 4μM) of V(IV)-Cit hybrid material, for 24, 48 and 72 h, compared to the untreated samples (control). Significance levels were assessed as follows: * $p < 0.05$ (significant), ** $p < 0.01$ (highly significant), *** $p < 0.001$ (extremely significant) and **** $p \leq 0.0001$ (extremely significant).

Figure S4 Morphological studies of N2a58, after treatment with two different concentrations (100 μg/mL and 1 mg/mL) of *Cornus mas* L. extracts, for 24, 48 and 72 h, compared to the untreated samples (control).

Figure S5 Morphological studies of SH-SY5Y, after treatment with two different concentrations (100 μg/mL and 1 mg/mL) of *Cornus mas* L. extracts, for 24, 48 and 72 h, compared to the untreated samples (control).

Figure S6 Morphological studies of N2a58, after treatment with two different concentrations (100 μM and 1 mM) of Zn(II)-Cit, for 24, 48 and 72 h, compared to the untreated samples (control).

Figure S7 Morphological studies of SH-SY5Y, after treatment with two different concentrations (100 μM and 1 mM) of Zn(II)-Cit, for 24, 48 and 72 h, compared to the untreated samples (control).

Figure S8 Morphological studies of N2a58, after treatment with two different concentrations (1 μM and 10 μM) of V(IV)-Cit, for 24, 48 and 72 h, compared to the untreated samples (control).

Figure S9 Morphological studies of SH-SY5Y, after treatment with two different concentrations (500 nM and 1 μM) of V(IV)-Cit, for 24, 48 and 72 h, compared to the untreated samples (control).

Figure S10 Morphological studies of SH-SY5Y, after treatment with combination of the *Cornus mas* L. extracts 1 mg/mL and 100 μM Zn(II)-Cit or *Cornus mas* L. extract 500 μg/mL and 500 nM V(IV)-Cit for 24, 48 and 72 h, compared to the untreated samples (control).

Figure S11 Chemotacticity studies using wound-healing assay of SH-SY5Y, after treatment with two different concentrations of the *Cornus mas* L. extracts (100 μg/mL and 1 mg/mL) for 24, 48 and 72 h, compared to the untreated samples (control).

Figure S12 Chemotacticity studies using wound-healing assay of SH-SY5Y, after treatment with 100 μM of Zn(II)-Cit or 500 nM of V(IV)-Cit for 24, 48 and 72 h, compared to the untreated samples (control).

Figure S13 **A.** Survival percentage of N2a58 cells, after treatment with the *Cornus mas* L. extracts (500 μg/mL), V(IV) (500 nM) or the mixture of both, prior to or after treatment with H_2O_2 500 μM, with the corresponding controls, compared to the untreated sample (control).

B. Survival percentage of SH-SY5Y, after treatment with the *Cornus mas* L. extracts (1 mg/mL), V(IV)-Cit (500 nM) or the mixture of both, prior to or after treatment with H_2O_2 250 μM, with the corresponding controls, compared to the untreated sample (control). Significance levels were assessed as follows: * $p < 0.05$ (significant), ** $p < 0.01$ (highly significant), *** $p < 0.001$ (extremely significant) and **** $p \leq 0.0001$ (extremely significant).

Figure S14: Intracellular concentration of zinc in ppb after treatment with 100 μM of $ZnCl_2$ for 1, 24, 48, and 72 h. Significance levels were assessed as follows: * $p < 0.05$

(significant), ** $p < 0.01$ (highly significant), *** $p < 0.001$ (extremely significant) and **** $p \leq 0.0001$ (extremely significant).

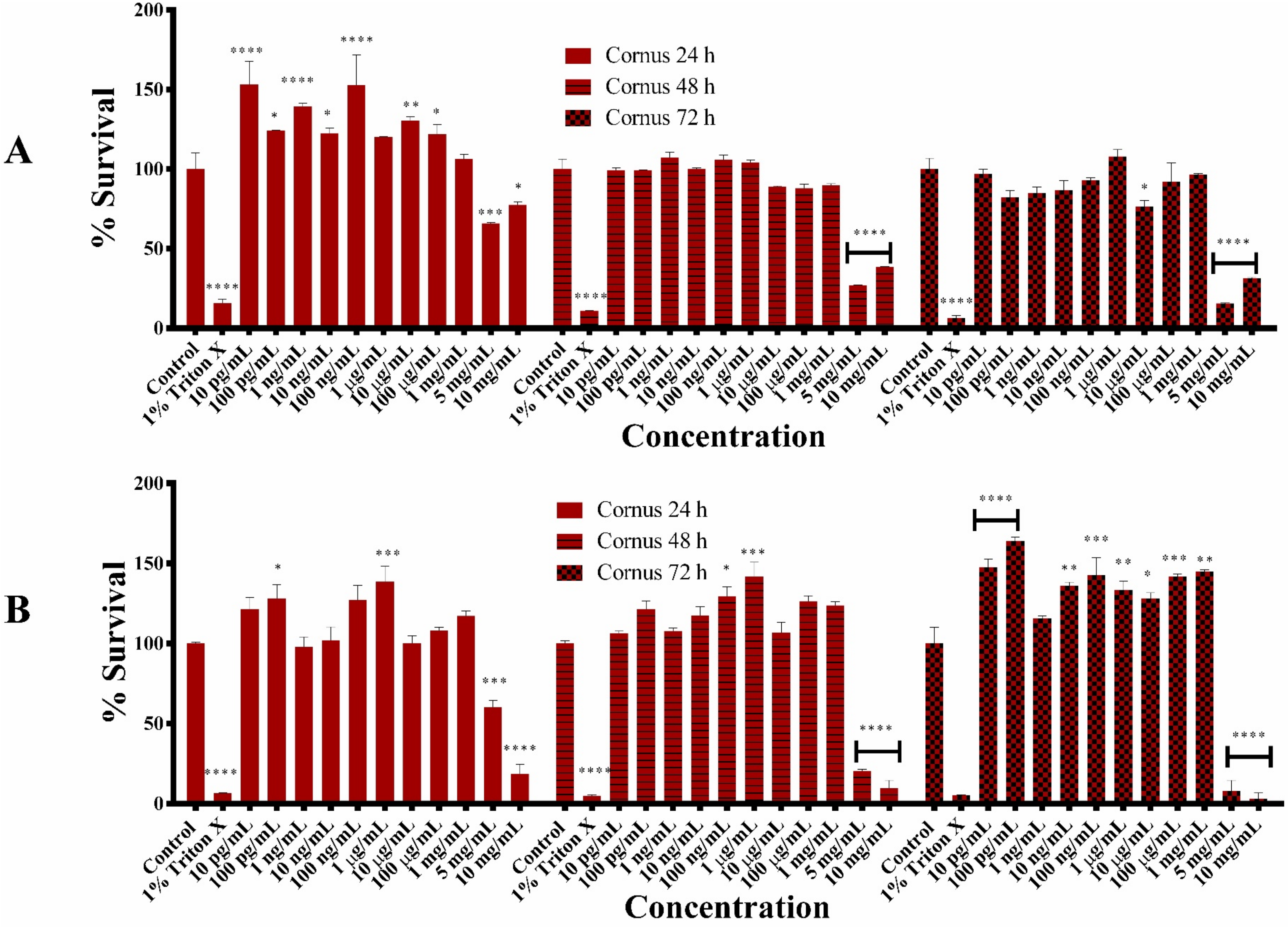
A
B
% Survival
Concentration
Cornus 24 h
Cornus 48 h
Cornus 72 h
0
50
100
150
200
Control
1% Triton X
10 pg/mL
100 pg/mL
1 ng/mL
10 ng/mL
100 ng/mL
1 µg/mL
10 µg/mL
100 µg/mL
1 mg/mL
5 mg/mL
10 mg/mL

Figure S1

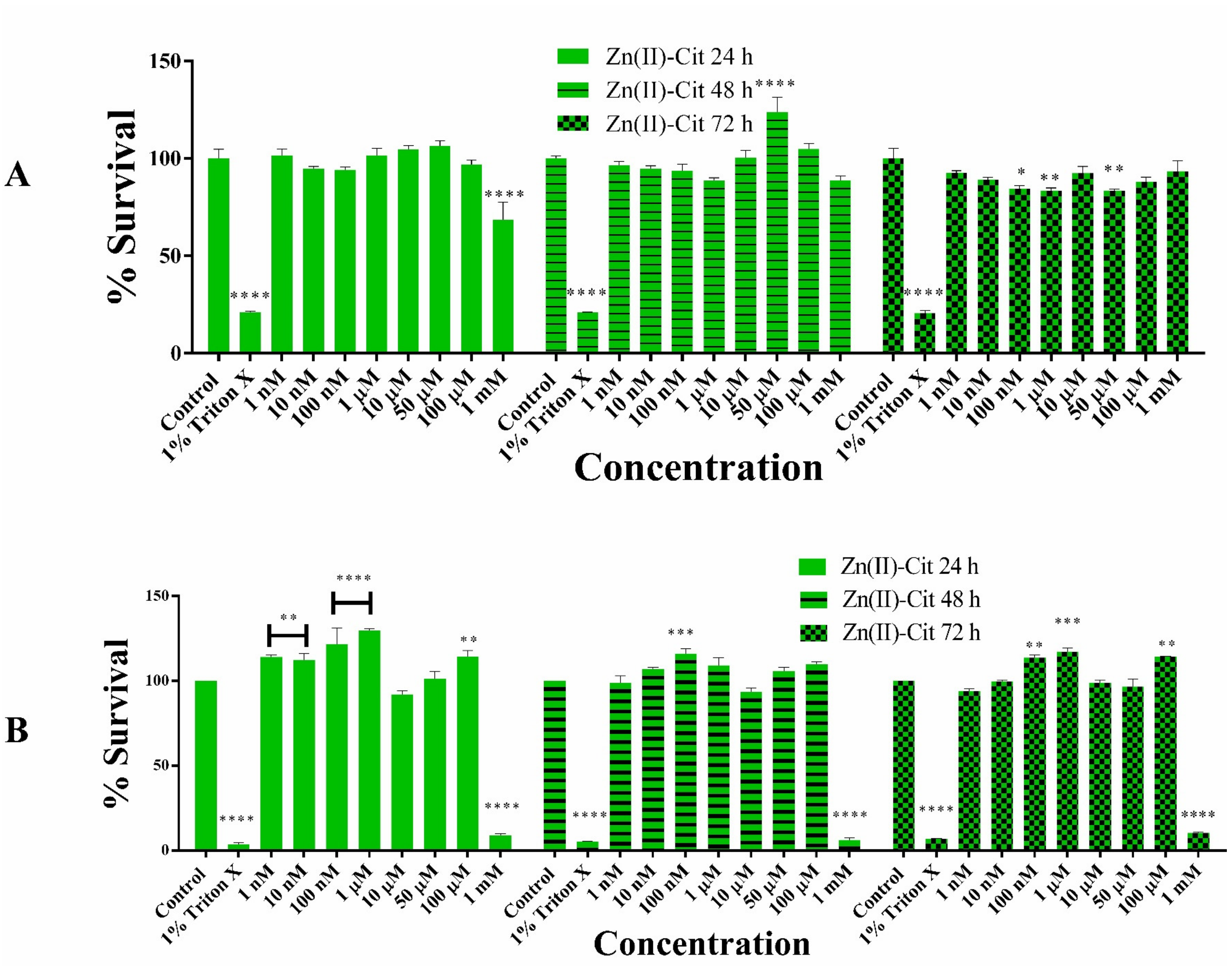
A
Zn(II)-Cit 24 h
Zn(II)-Cit 48 h
Zn(II)-Cit 72 h
% Survival
Concentration
Control
1% Triton X
1 nM
10 nM
100 nM
1 µM
10 µM
50 µM
100 µM
1 mM
B
Zn(II)-Cit 24 h
Zn(II)-Cit 48 h
Zn(II)-Cit 72 h
% Survival
Concentration

Figure S2

A

B

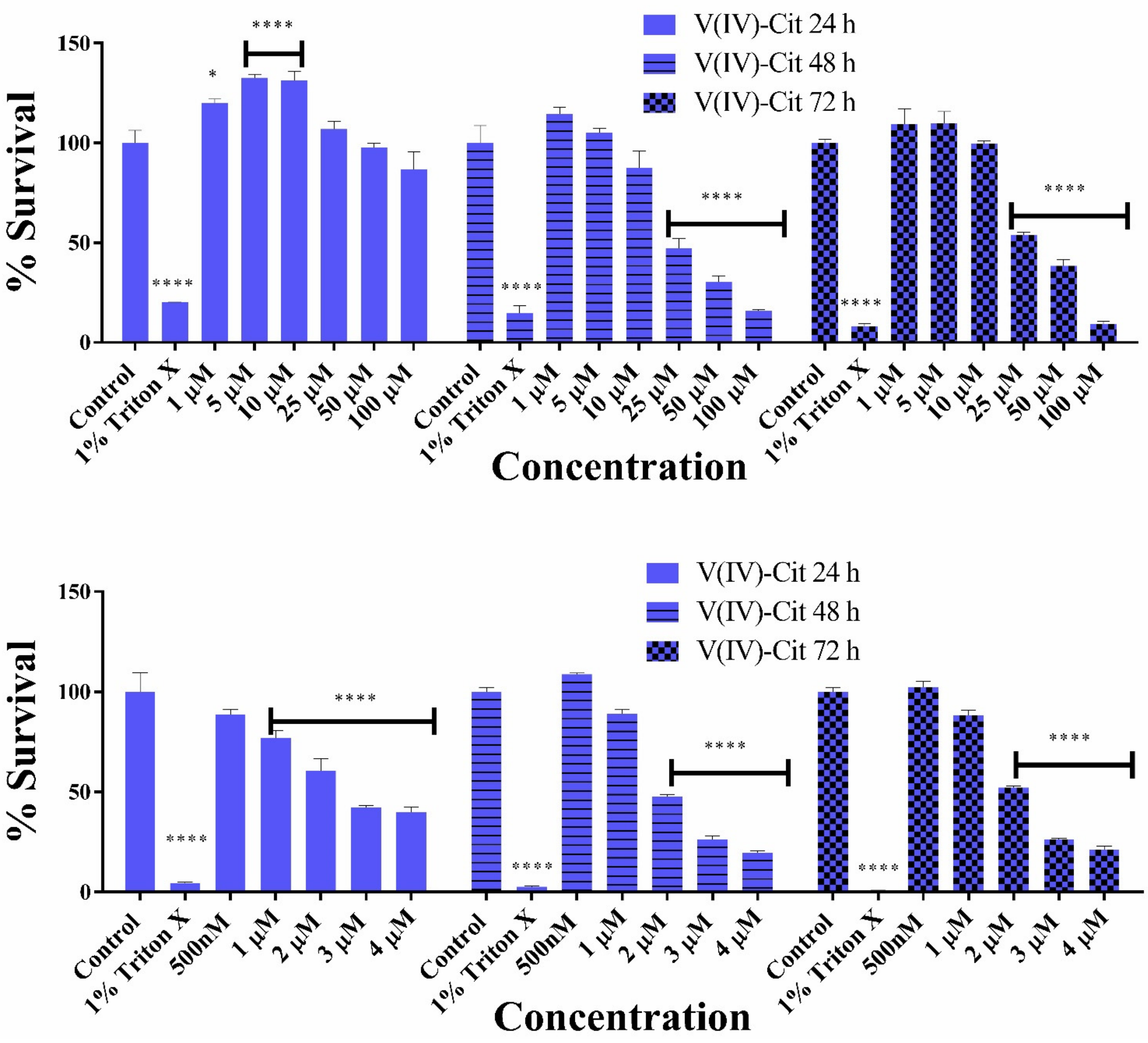

V(IV)-Cit 24 h
V(IV)-Cit 48 h
V(IV)-Cit 72 h
% Survival
Concentration
Control
1% Triton X
1 μM
5 μM
10 μM
25 μM
50 μM
100 μM
500nM
2 μM
3 μM
4 μM
****
*

Figure S3

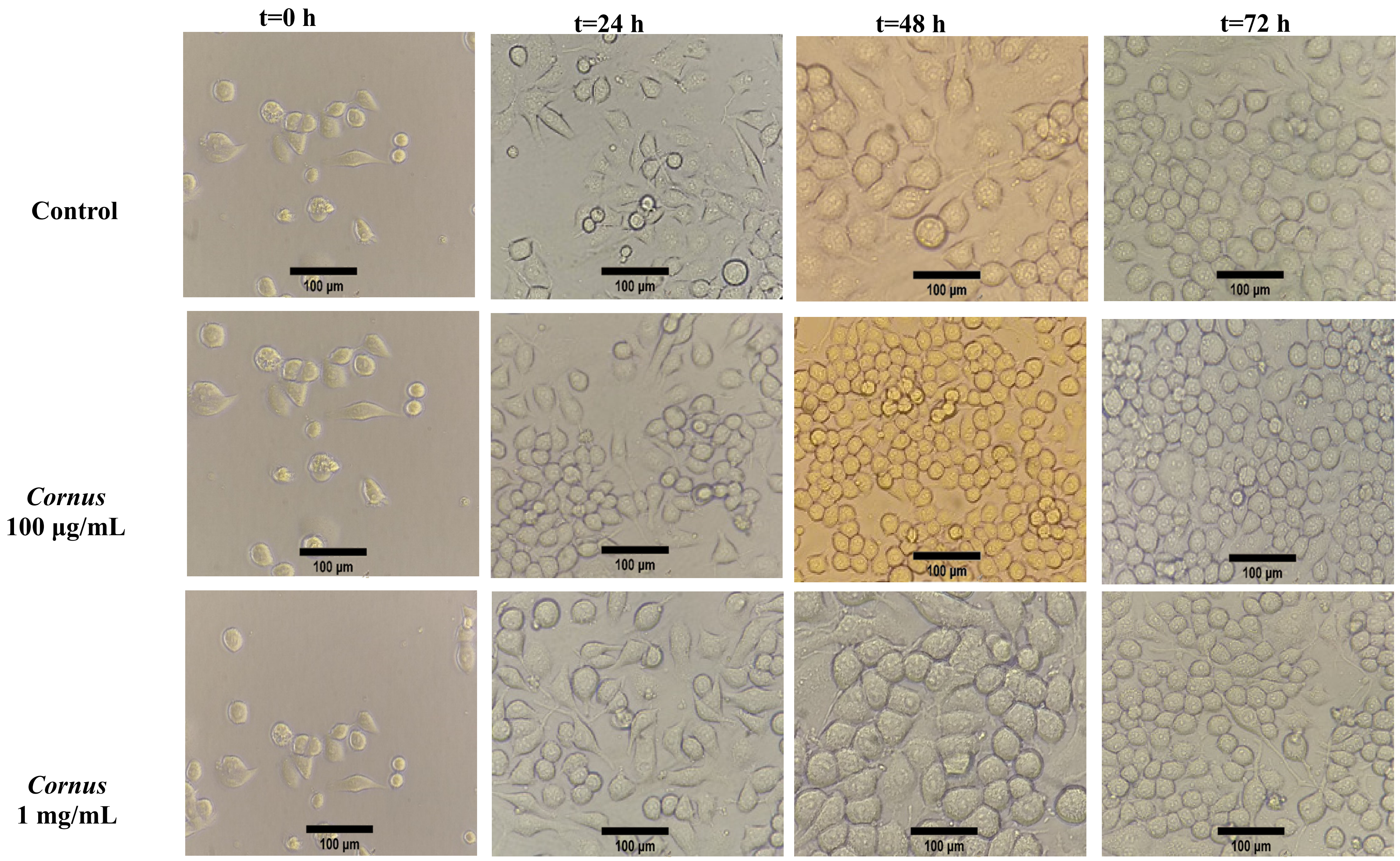
t=0 h
t=24 h
t=48 h
t=72 h
Control
Cornus
100 µg/mL
Cornus
1 mg/mL
100 µm

Figure S4

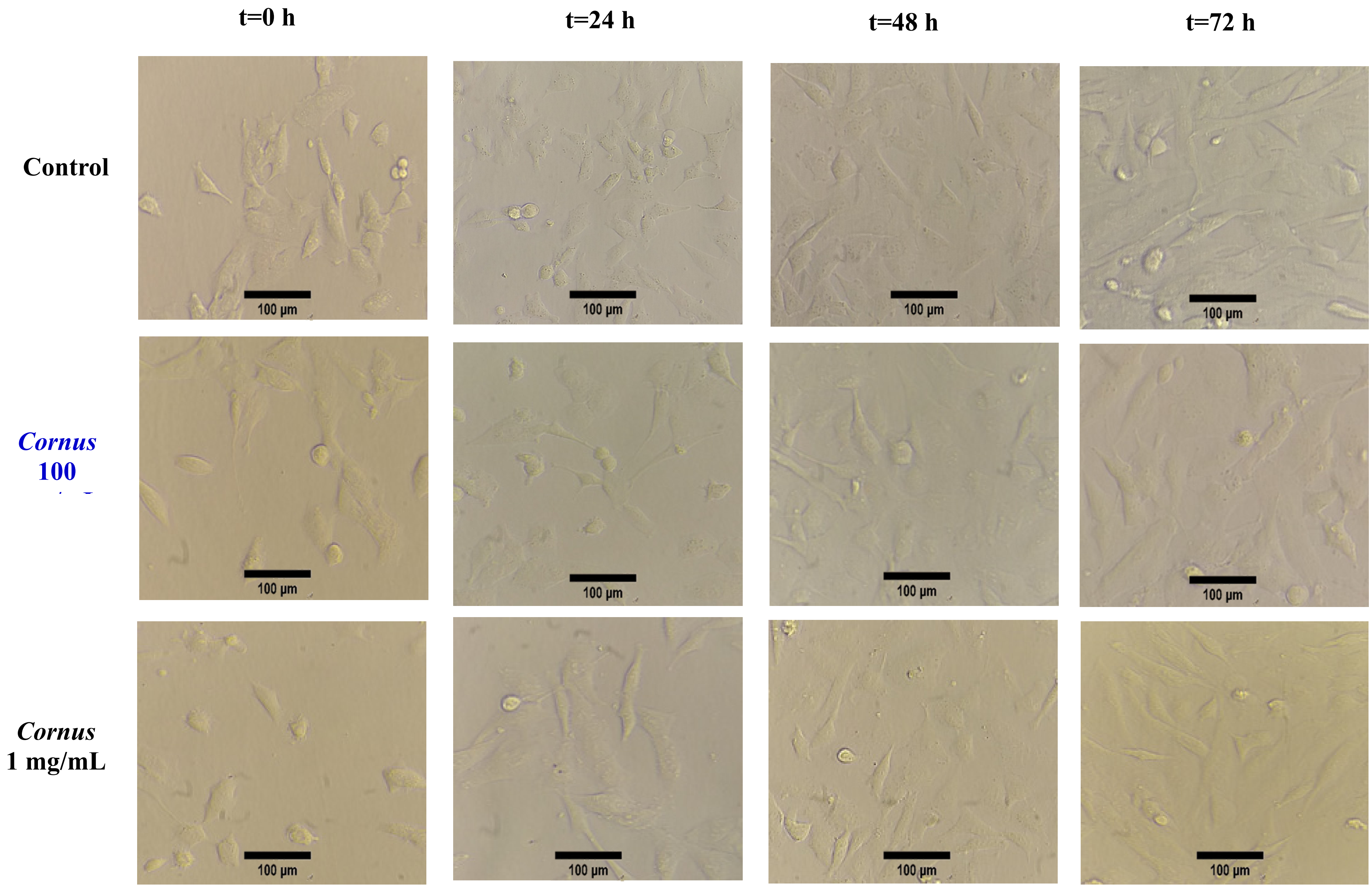

Figure S5

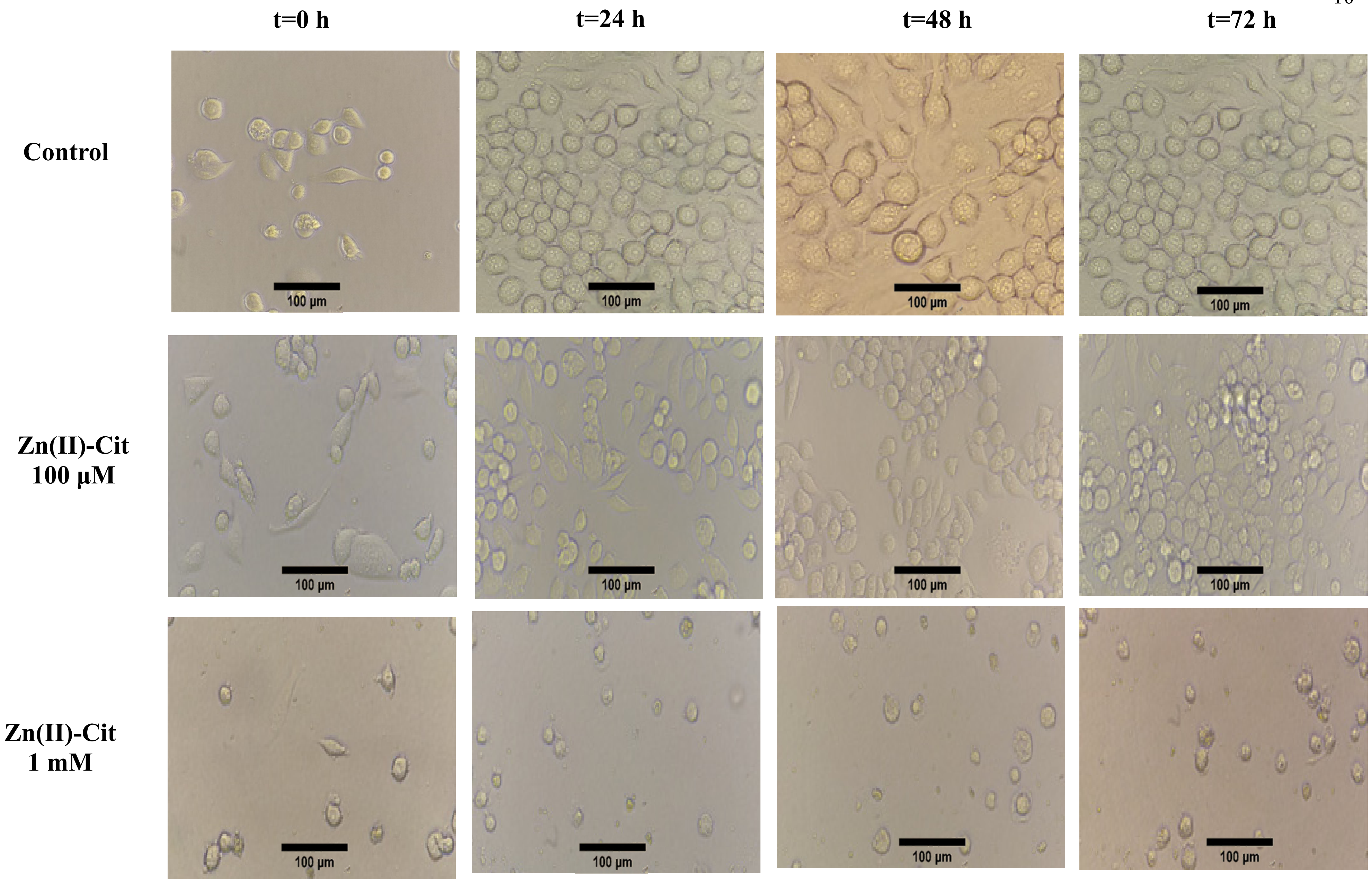

Figure S6

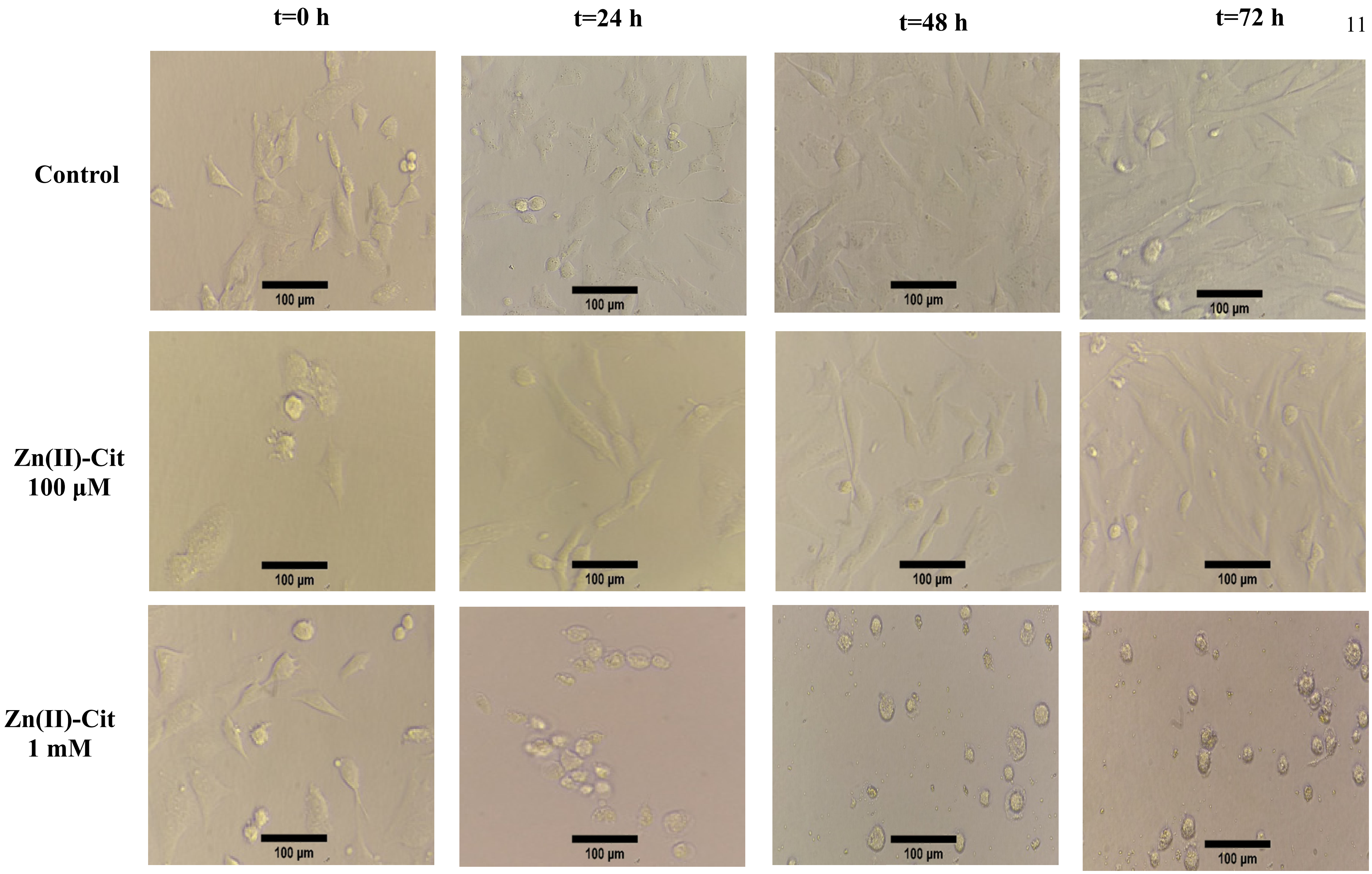

Figure S7

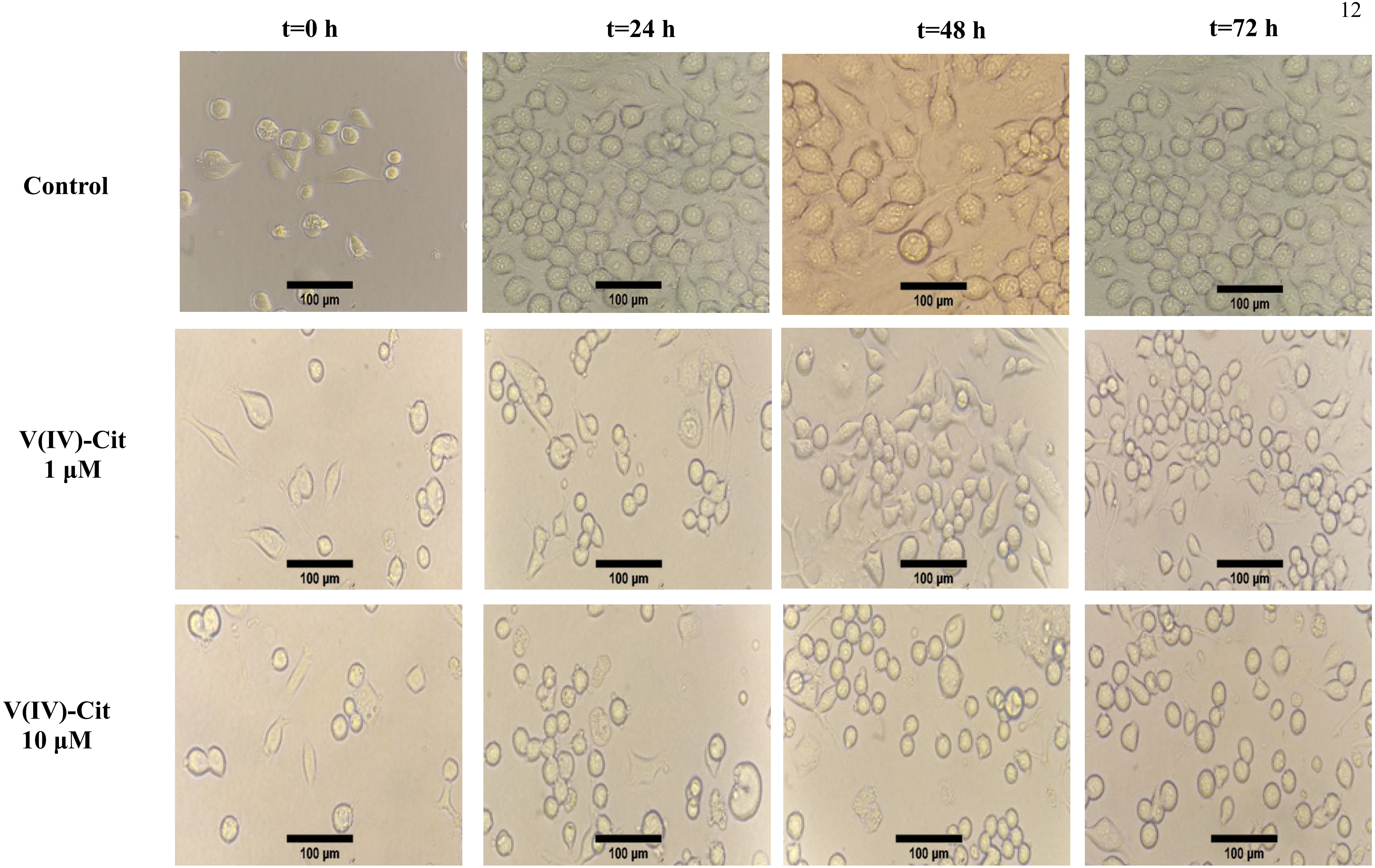

Figure S8

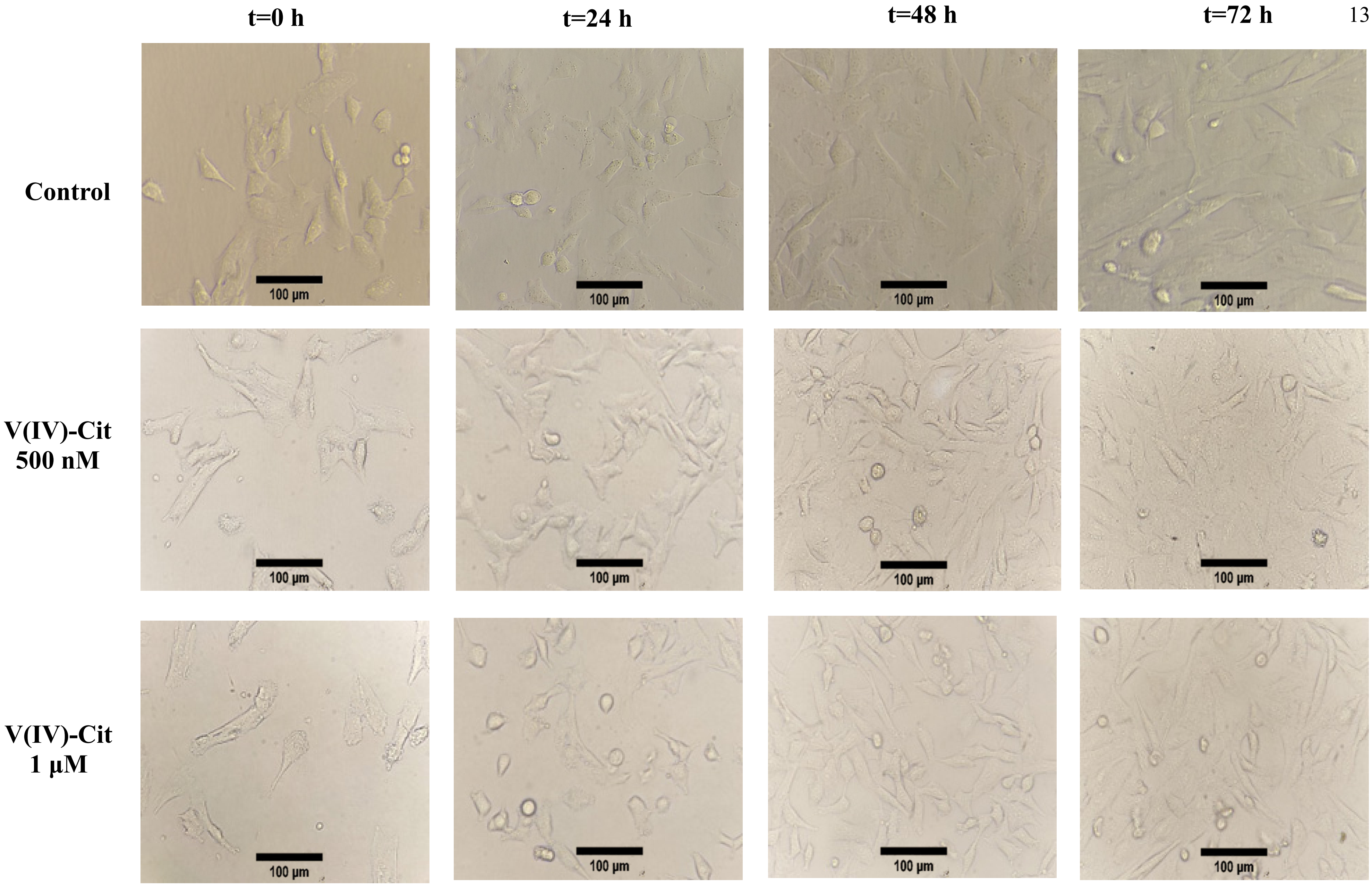

Figure S9

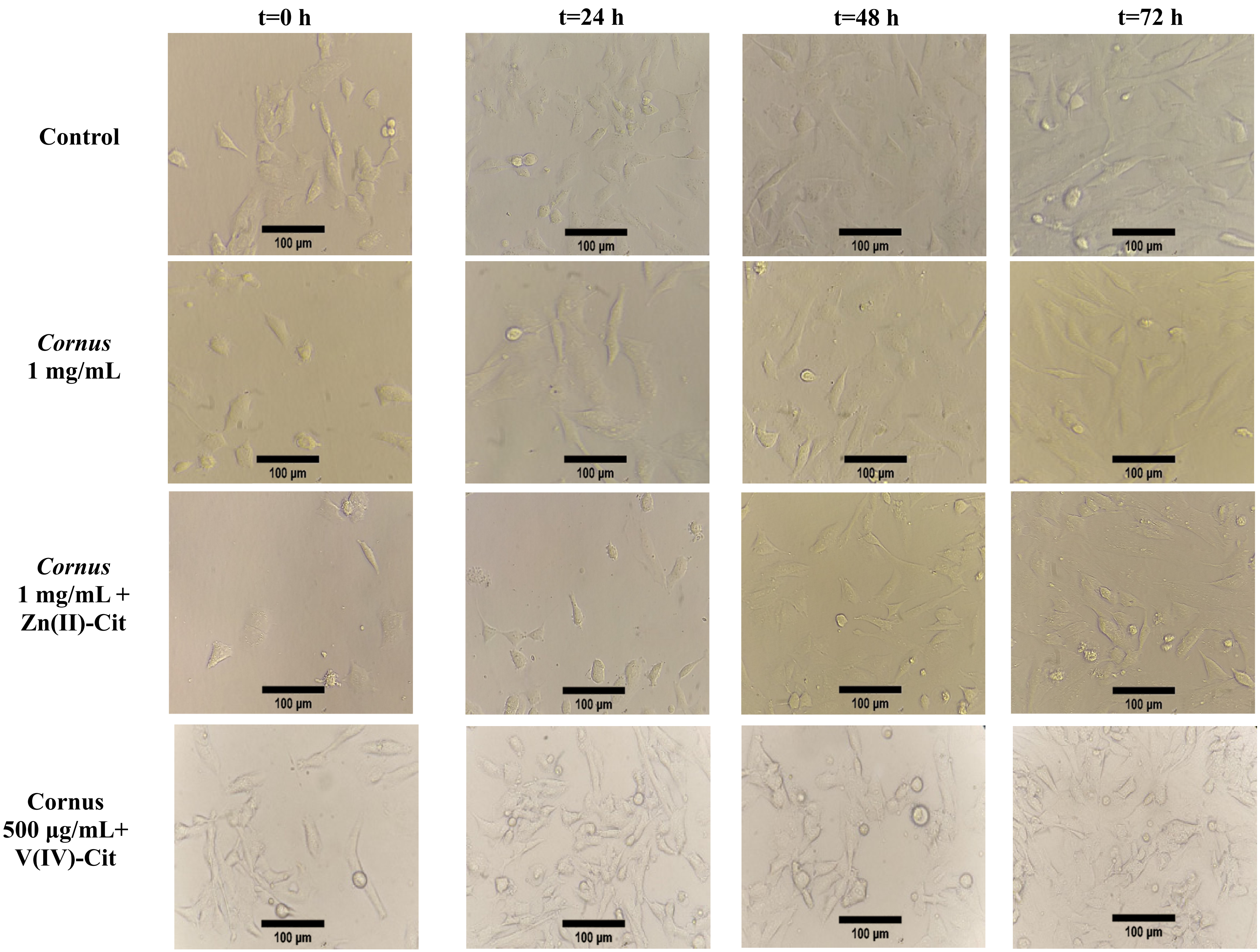
t=0 h
t=24 h
t=48 h
t=72 h
Control
Cornus
1 mg/mL
Cornus
1 mg/mL +
Zn(II)-Cit
Cornus
500 µg/mL+
V(IV)-Cit
100 µm

Figure S10

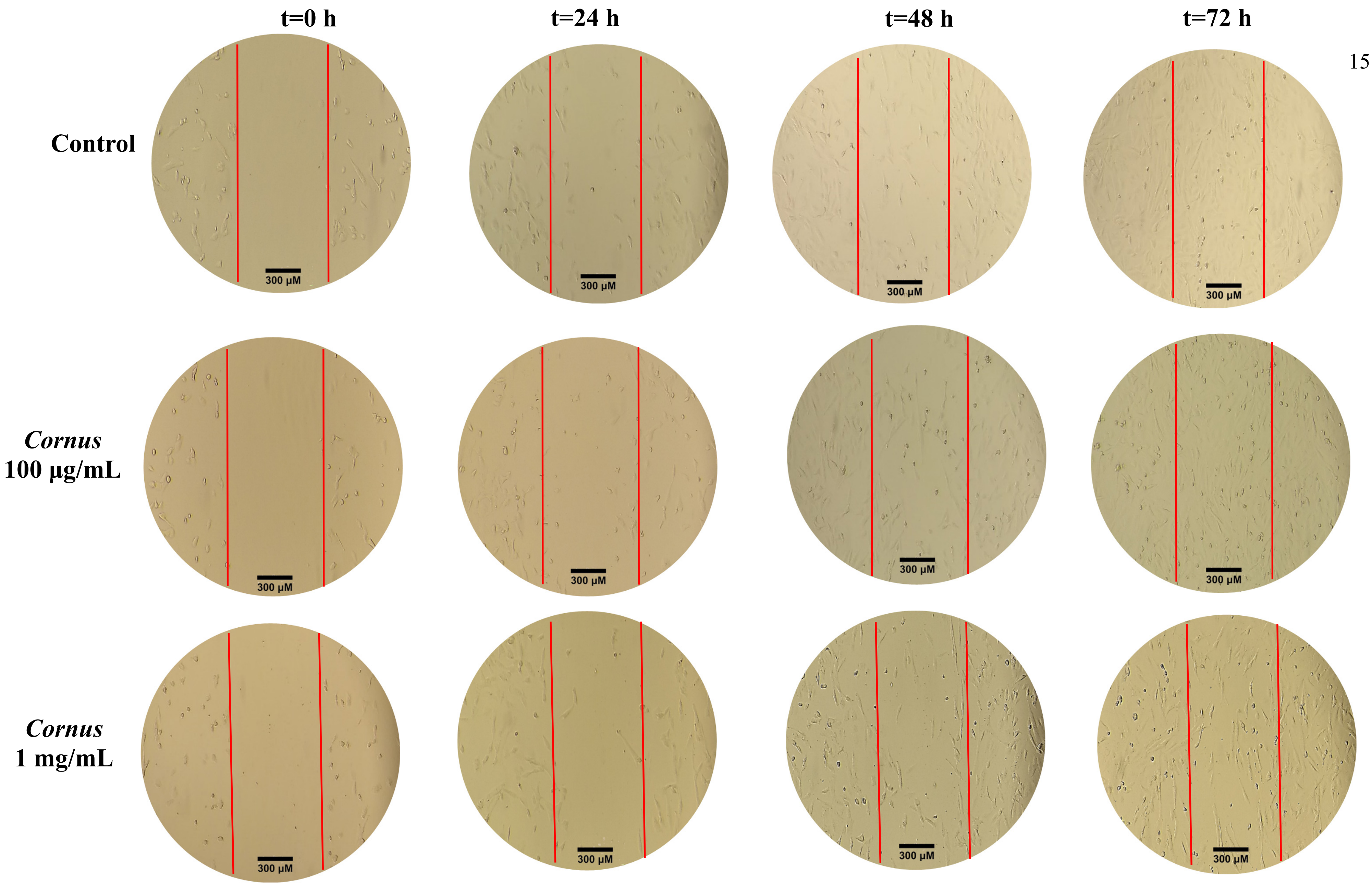
t=0 h
t=24 h
t=48 h
t=72 h
Control
Cornus
100 µg/mL
Cornus
1 mg/mL
300 µM

Figure S11

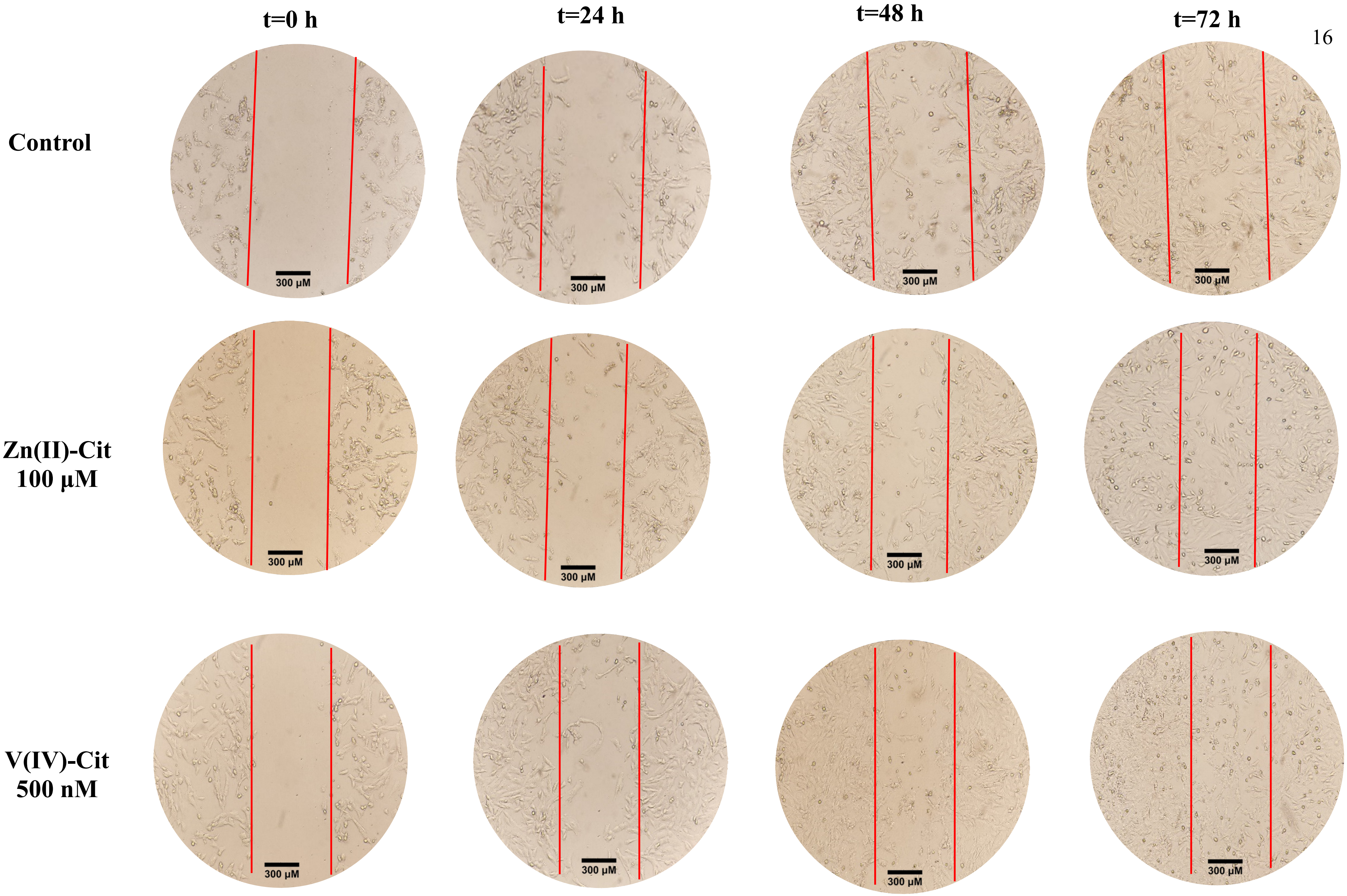
t=0 h
t=24 h
t=48 h
t=72 h
Control
Zn(II)-Cit 100 µM
V(IV)-Cit 500 nM
300 µM

Figure S12

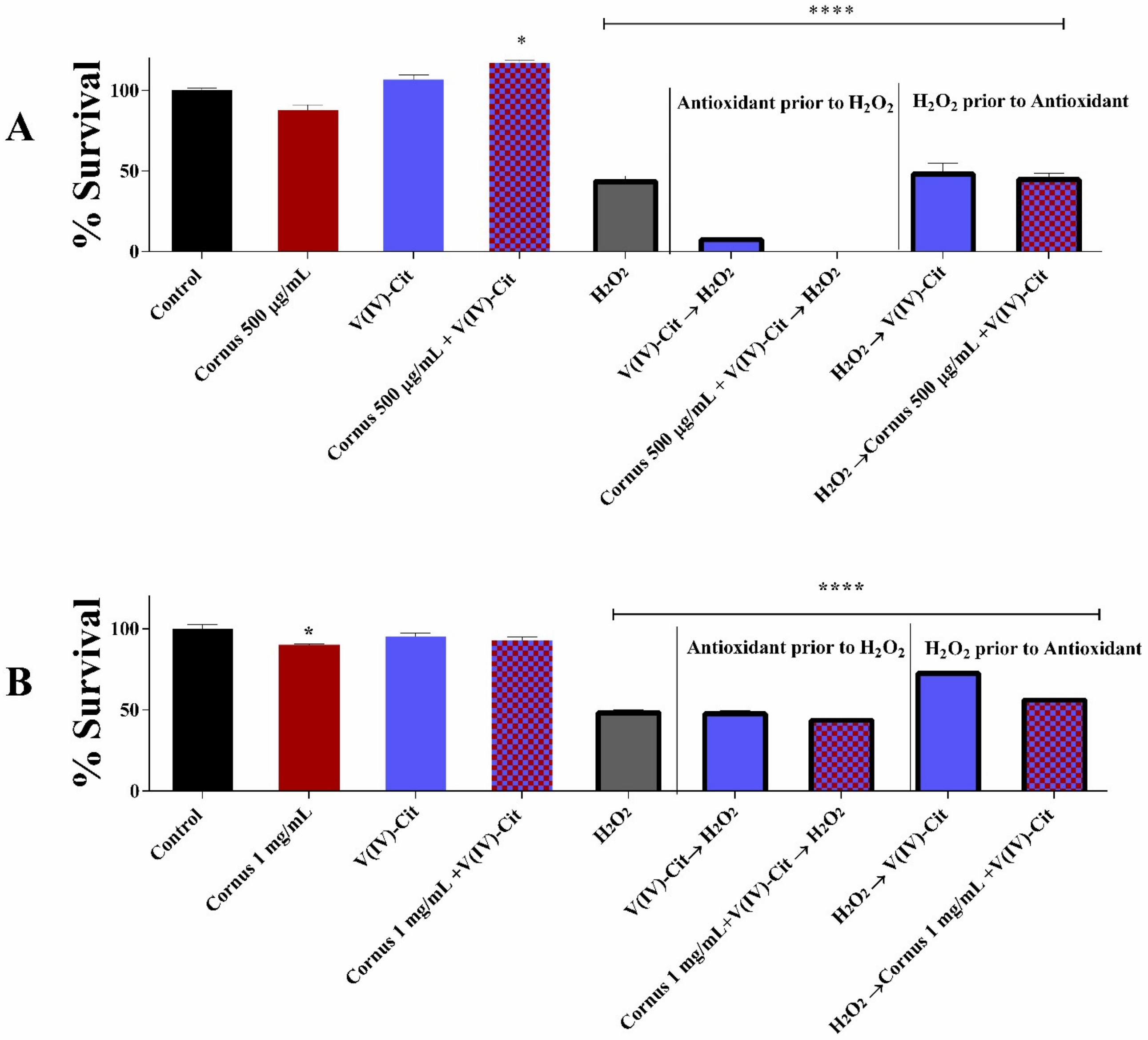
A
% Survival
100
50
0
****
*
Antioxidant prior to H_2O_2
H_2O_2 prior to Antioxidant
Control
Cornus 500 µg/mL
V(IV)-Cit
Cornus 500 µg/mL + V(IV)-Cit
H_2O_2
V(IV)-Cit → H_2O_2
Cornus 500 µg/mL + V(IV)-Cit → H_2O_2
H_2O_2 → V(IV)-Cit
H_2O_2 →Cornus 500 µg/mL +V(IV)-Cit
B
% Survival
100
50
0
****
*
Antioxidant prior to H_2O_2
H_2O_2 prior to Antioxidant
Control
Cornus 1 mg/mL
V(IV)-Cit
Cornus 1 mg/mL +V(IV)-Cit
H_2O_2
V(IV)-Cit→ H_2O_2
Cornus 1 mg/mL+V(IV)-Cit → H_2O_2
H_2O_2 → V(IV)-Cit
H_2O_2 →Cornus 1 mg/mL +V(IV)-Cit

Figure S13

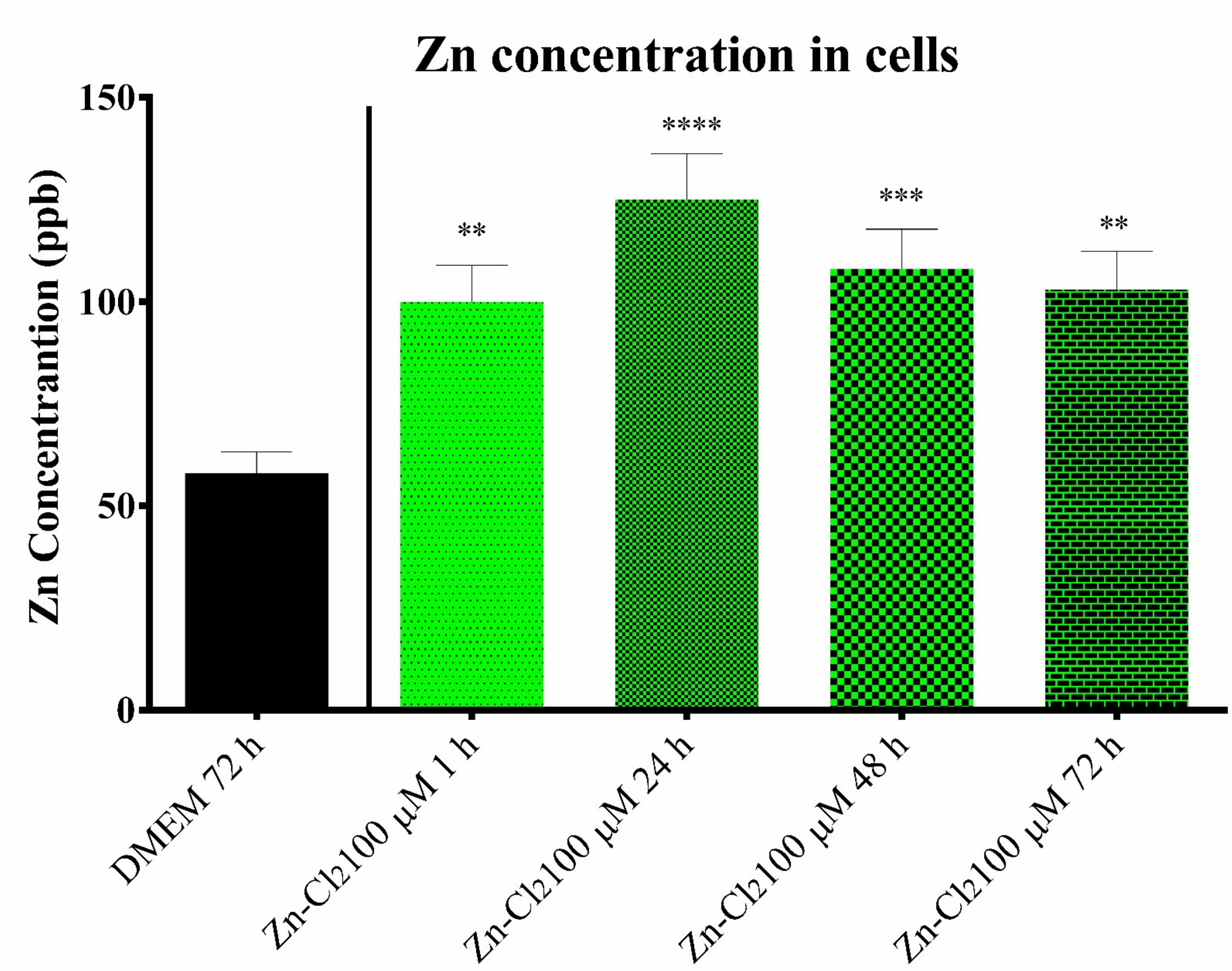
Zn concentration in cells
Zn Concentrantion (ppb)
150
100
50
0
****
**
***
**
DMEM 72 h
Zn-Cl2100 μM 1 h
Zn-Cl2100 μM 24 h
Zn-Cl2100 μM 48 h
Zn-Cl2100 μM 72 h

Figure S14
